# Neuroimaging-Derived Biomarkers of the Antidepressant Effects of Ketamine

**DOI:** 10.1016/j.bpsc.2022.11.005

**Published:** 2022-11-26

**Authors:** Artemis Zavaliangos-Petropulu, Noor B. Al-Sharif, Brandon Taraku, Amber M. Leaver, Ashish K. Sahib, Randall T. Espinoza, Katherine L. Narr

**Affiliations:** Ahmanson-Lovelace Brain Mapping Center, Department of Neurology, David Geffen School of Medicine, University of California, Los Angeles, Los Angeles, California (AZ-P, NBA-S, BT, AKS, KLN); Department of Radiology, Northwestern University, Chicago, Illinois (AML); and the Jane and Terry Semel Institute for Neuroscience and Human Behavior, Department of Psychiatry and Biobehavioral Sciences, David Geffen School of Medicine, University of California, Los Angeles, Los Angeles, California (RTE, KLN).

## Abstract

Major depressive disorder is a highly prevalent psychiatric disorder. Despite an extensive range of treatment options, about a third of patients still struggle to respond to available therapies. In the last 20 years, ketamine has gained considerable attention in the psychiatric field as a promising treatment of depression, particularly in patients who are treatment resistant or at high risk for suicide. At a subanesthetic dose, ketamine produces a rapid and pronounced reduction in depressive symptoms and suicidal ideation, and serial treatment appears to produce a greater and more sustained therapeutic response. However, the mechanism driving ketamine’s antidepressant effects is not yet well understood. Biomarker discovery may advance knowledge of ketamine’s antidepressant action, which could in turn translate to more personalized and effective treatment strategies. At the brain systems level, neuroimaging can be used to identify functional pathways and networks contributing to ketamine’s therapeutic effects by studying how it alters brain structure, function, connectivity, and metabolism. In this review, we summarize and appraise recent work in this area, including 51 articles that use resting-state and task-based functional magnetic resonance imaging, arterial spin labeling, positron emission tomography, structural magnetic resonance imaging, diffusion magnetic resonance imaging, or magnetic resonance spectroscopy to study brain and clinical changes 24 hours or longer after ketamine treatment in populations with unipolar or bipolar depression. Though individual studies have included relatively small samples, used different methodological approaches, and reported disparate regional findings, converging evidence supports that ketamine leads to neuroplasticity in structural and functional brain networks that contribute to or are relevant to its antidepressant effects.

About 50% of patients with major depressive disorder (MDD) do not respond to first-line monoaminergic antidepressants (1) and after multiple treatment failures are characterized as having treatment-resistant depression (2). At a subanesthetic dose, ketamine produces profound and rapid reductions of depression symptoms (3–5) and suicidality (6) in over 60% of patients with MDD (4,7). Though symptom relief is typically transient (<1 week), larger and more sustained antidepressant response and remission occur with serial ketamine therapy (8).

In vivo studies of brain structure, function, connectivity, and metabolism can help discern the functional pathways and systems contributing to ketamine’s therapeutic effects at the macro scale (9,10) and may inform more effective individualized treatment approaches. Here, we synthesize findings identified using PRISMA (Preferred Reporting Items for Systematic Reviews and Meta-Analyses) methods (11) from neuroimaging studies in MDD published in English from 2011 to 2022 that investigated the effects of ketamine on functional and structural brain systems or imaging biomarkers predictive of ketamine antidepressant response. Original research articles that collected brain imaging and clinical outcomes ≥24 hours post ketamine administration in adult populations with unipolar or bipolar depression were included. Preclinical studies addressing the molecular and cellular mechanisms of ketamine (12,13) or neuroimaging studies that investigated the effects of ketamine only during or shortly after (<24 hours) ketamine treatment were excluded (14–19). Figure 1 shows the search methods and inclusion criteria.

## FUNCTIONAL MAGNETIC RESONANCE IMAGING

### Resting-State Functional Magnetic Resonance Imaging

Extensive neuroimaging evidence supports that the pathophysiology of MDD includes the dysregulation of multiple large-scale brain networks. For example, connectivity within or between the default mode network (DMN), salience network (SN), cingulo-opercular network (CON), frontoparietal network (FPN)/central executive network (CEN), orbito-affective network (OAN), and ventromedial affective network (VMN) are repeatedly implicated in depression and appear to influence treatment outcomes (20–23). These networks and their nodes are pivotal for emotion processing; autonomic responses to emotion, memory, and social cognition (hippocampus, amygdala, septal area, anterior thalamus, and hypothalamus); higher cognition; motivation and mood regulation (dorsal anterior cingulate cortex [dACC] and subgenual ACC [sgACC] and dorsolateral prefrontal cortex [DLPFC] and parietal association regions); reward processing (ventral striatum, habenula); and vegetative states (midbrain/brainstem structures) (22–24). Accordingly, the majority of recent ketamine imaging studies have used blood oxygen level–dependent (BOLD) resting-state functional magnetic resonance imaging (rsfMRI) to measure the temporal coherence of intrinsic brain activity across different functional brain systems in the absence of a specific task (25,26). Network connectivity can be measured in many different ways with rsfMRI, each having its limitations (27–29). In this review, we report rsfMRI findings for large-scale cortical networks and nodes and for whole brain and global brain connectivity (Table 1).

#### Large-Scale Cortical Networks and Nodes.

Several independent rsfMRI studies have reported changes in resting-state functional connectivity (RSFC) in MDD in the days following ketamine treatment. The DMN, engaged during introspective activities and consisting of anterior/dorsal (medial PFC, precuneus, and inferior parietal cortex) and ventral (hippocampus/parahippocampus) subsystems, is perhaps the most widely studied large-scale network in depression (21). Several studies have reported ketamine-related changes in DMN RSFC. For example, 2 weeks following a continuous 96-hour intravenous (IV) ketamine infusion (0.6 mg/kg/hour), RSFC decreased within ventral DMN limbic nodes but increased between subcortical and cortical DMN/CON nodes (30). However, these patterns did not vary based on ketamine response. A separate study similarly observed increased RSFC between the DMN and the insula, a key node of the CON and SN, as well as with frontal, parietal, and occipital cortices 2 days after single IV ketamine infusion compared with placebo in MDD (31). Here, increased DMN–insula connectivity normalized toward control levels 2 days post ketamine but reversed 10 days following treatment. Another study found when adding ketamine to propofol-electroconvulsive treatment, RSFC within the DMN decreased (32).

The FPN/CEN, comprising of dorsal and ventral frontoparietal subsystems, is a large resting-state network active during attention, cognitive states, and emotion regulation that is frequently linked with depression. Also implicated in ketamine MRI studies, decreased RSFC has been reported within the frontal component of the CEN 48 hours following single-dose ketamine in PFC and dACC seed-based analysis (33). Negative and positive correlations with suicidal ideation were observed for RSFC between the left and right dACC and DLPFC–left superior parietal cortex for 0.5 mg/kg and 0.2 mg/kg doses, respectively. After explicitly examining RSFC between the hippocampus and amygdala with the DMN, FPN/CEN, and SN, increased and normalized RSFC was observed between the right amygdala and the right CEN 24 hours after patients received 4 serial ketamine infusions (34). Further, decreased left amygdala–SN RSFC associated with improved behavioral inhibition, while negative connectivity between the right hippocampus and the left CEN correlated with improved anhedonia (34,35).

The sgACC, considered a node of the VMN and reciprocally connected with limbic, ventral striatal, habenular, and thalamic structures, is involved in modulating emotion and reward response (36) and linked with antidepressant response following behavioral, pharmacological, and brain stimulation interventions (20). Increased RSFC between the sgACC, caudate, and insula was observed 2 weeks after single 96-hour ketamine IV treatment (30), though RSFC between the sgACC and DMN decreased. At least 2 studies have linked sgACC connectivity with overall antidepressant response post ketamine. Specifically, a multisite study reported associations between depression symptom improvement (37,38) and increased RSFC between the sgACC and supplementary motor area and the sgACC and DLPFC 24 hours following single IV ketamine treatment (39). However, another study found that symptom improvement (38) was associated with lower RSFC between the sgACC and right amygdala (38,40). Lower sgACC–right amygdala RSFC at baseline was also found to be present in patients subsequently identified as treatment responders (40).

Prior data also suggest that frontostriatal circuitry influences ketamine response. Here, lower PFC–striatal RSFC at baseline has been found to associate with subsequent improvements in depressive symptoms (37,41). Additionally, a seed-based analysis showing increased frontostriatal connectivity 2 days after single IV ketamine treatment (42) found increased connectivity between the VLPFC and caudate to associate with improved anhedonia (35).

The habenula, part of the epithalamus with direct connections to limbic structures and the basal ganglia, plays a role in emotion, reward, and motivation (43). Increased RSFC has been reported between the habenula and right DLPFC, which has been associated with improved antidepressant response (38) 24 hours after single IV ketamine treatment (44). Further, increased RSFC between the right habenula and right and left occipital cortex, right temporal pole, and right parahippocampal gyrus associated with improved subjective mood ratings (45).

#### Whole Brain and Global Brain Connectivity.

The larger literature suggests that several interacting brain networks underlie the pathophysiology of depression and that multiple functional networks contribute to antidepressant response (23). Prior ketamine rsfMRI studies have mostly focused on targeting major networks or components thereof, potentially missing other changes in the functional connectome. To address this limitation, one study employed a data-driven whole brain approach before and 24 hours after serial ketamine therapy (46) to show that RSFC within and between the somatomotor network, FPN/CEN, and visual network normalized with treatment and distinguished control subjects from patients at baseline. This study also showed a normalization of circuitry between the cerebellum, SN, and striatum, which correlated with antidepressant response (37).

Global brain connectivity (GBC), which measures the connectivity strength between each voxel to all other gray matter voxels, has been investigated in 4 ketamine studies. One study (47) found ketamine-related GBC increases in the PFC and reduced GBC in the cerebellum. Treatment responders showed greater GBC changes in the lateral PFC, caudate, and insula 24 hours post single-dose ketamine. Follow-up seed-based analysis suggested that ketamine reduced hyper-connectivity within the PFC and enhanced hypoconnectivity between the PFC and other brain regions. In a larger sample, the same investigators found an increase in PFC GBC regression (GBCr) 24 hours after ketamine when compared with placebo and baseline (48,49), which negatively correlated with symptom improvement. However, a separate study (50) did not find significant changes to GBC when neuroimaging was performed 2 to 3 days post ketamine. Finally, a study investigating GBC density after serial ketamine in treatment-resistant bipolar depression (51) found decreased density in the bilateral insula, right caudate, and bilateral VLPFC and increased density in the bilateral postcentral gyrus, sgACC, thalamus, and cerebellum. These changes appeared 24 hours post ketamine, peaked at 1 week, and diminished by the third week; significant associations with clinical measures were not detected.

#### Summary.

**Figure 2.**
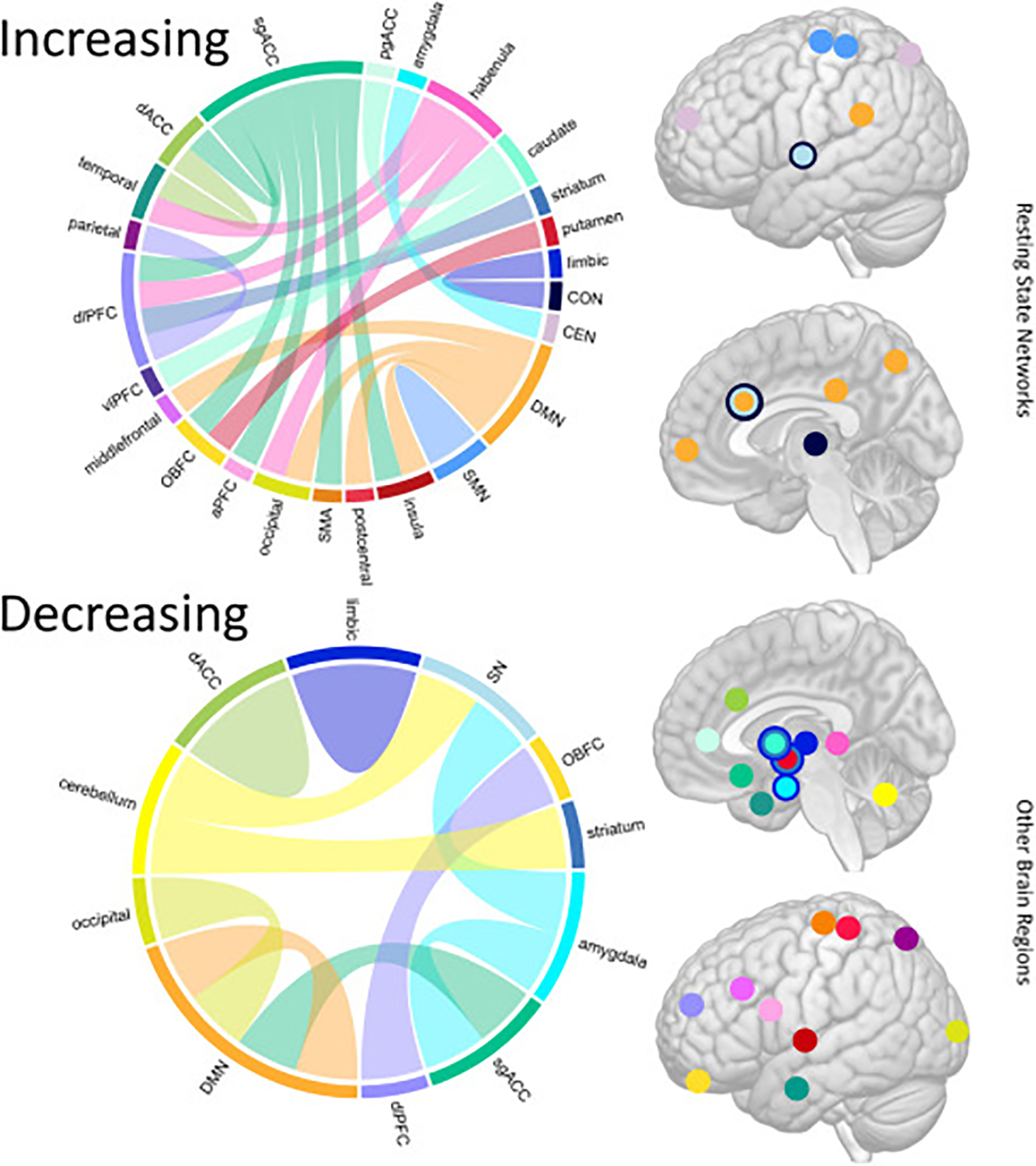
This illustration synthesizes results across functional magnetic resonance imaging studies that have addressed the effects of subanesthetic ketamine on resting-state functional connectivity in patients with major depression. Here, connectograms are used to describe increasing (top) and decreasing (bottom) changes in resting-state functional connectivity between specific brain regions and resting-state networks observed across studies following ketamine treatment. Corresponding atlases that are color coordinated with the resting-state networks (top) and brain regions (bottom) represented in the connectograms are shown on the right. Table S1 details the connections and their corresponding references. aPFC, anterior prefrontal cortex; CEN, central executive network; CON, cingulo-opercular network; dACC, dorsal anterior cingulate cortex; dlPFC, dorsolateral prefrontal cortex; DMN, default mode network; OBFC, orbitofrontal cortex; pgACC, pregenual anterior cingulate cortex; sgACC, subgenual anterior cingulate cortex; SMA, sensorimotor area; SMN, somatomotor network; SN, salience network; vlPFC, ventrolateral prefrontal cortex.

Subanesthetic ketamine leads to plasticity in multiple resting-state networks and their components (Figure 2). Because existing investigations have used different analysis approaches (e.g., theory-driven, seed-based vs. data-driven, whole-brain analysis methods), sample sizes are typically small and have overlapped among reports, and study designs (open-label, randomized controlled trial, post ketamine assessment time points) and clinical populations vary, convergence among findings remains relatively low. However, this work still provides important insight and future leads concerning the influence of subanesthetic ketamine on functional brain circuity and clinical outcomes. For example, existing data support that ketamine leads to decreased activity within the DMN (20,34,35) and within the FPN/CEN (24), potentially affecting behaviors associated with negatively biased self-referential processing and executive functions/emotional regulation in depression, respectively. When generalizing findings, RSFC instead appears to increase between different large-scale cortical networks such as the DMN and FPN/CEN with nodes including the sgACC, anterior insula, striatum, amygdala, and habenula (30,39,42,44,45,47). Together, these patterns suggest that ketamine modulates circuitry that may be both under- and overreactive prior to treatment. Data-driven results also emphasize the contribution of sensory systems and cortico-striatal-cerebellar loops that encompass the SN as a potential biomarker for ketamine response (46,47). Notably, individual studies have shown that changes in RSFC in nodes including the amygdala, sgACC, and habenula to other subcortical or higher cortical association regions relate to clinical response (39,40,44). Changes in limbic RSFC (amygdala, hippocampus) have also been linked to improved behavioral inhibition (34), anhedonia (34,42), and suicidality (33).

### Task-based fMRI

Disturbances in emotion regulation and processing and other cognitive functions linked with depression can be probed by examining the fMRI BOLD signal as participants actively engage in functional tasks (52–54). Here, we summarize findings from ketamine studies using task-based fMRI organized by brain activation task, including response inhibition, reward processing, emotional judgment, and emotional face recognition (Table 2).

Two fMRI studies probed changes in response inhibition (NoGo>Go) as a proxy for cognitive control (55,56). Decreased activation in the inferior PFC/DLPFC and parietal regions, right cerebellum, and visual cortex were observed during response inhibition post serial ketamine treatment, which normalized toward healthy control subjects (55). Decreased activation in prefrontal-parietal inhibitory control networks and motor and insular regions 24 hours post single and serial treatment were associated with and predicted subsequent improvements in mood. In an overlapping sample, investigators used psycho-physiological interaction models to interrogate relationships between the cerebellum and FPN, somatomotor network, and SN (56). Results revealed significant decreases in psycho-physiological interaction model connectivity between the cerebellum, FPN, and somatomotor network in remitters only. Baseline values of cerebellar-FPN and cerebellar-SN psycho-physiological interaction models were associated with clinical outcomes (45).

MDD is linked with dysfunctional reward processing, including motivation, reinforcement learning, and hedonic capacity (57,58). A study using an incentive flanker task showed reduced sgACC activation to positive and negative feedback 5 days following single-dose ketamine, normalizing toward healthy control subjects (59). Greater pre-ketamine sgACC hyperactivity to positive feedback also associated with larger posttreatment improvements in anhedonia. Using a modified monetary incentive delay task, an independent study found increased insula and orbitofrontal activation during the anticipatory phase of reward 24 hours post ketamine (60). Increased ventral striatum and orbitofrontal cortex activity involved in reward processing (61,62) [often hypoactive in MDD (63)] was observed 7 days postinfusion.

Several studies have used variants of the emotion recognition task to probe ketamine’s effects on emotion networks. Using the emotional judgment task, during which participants rate the emotional valence of pictures, decreased amygdala and insula response was observed during negatively valanced picture ratings 24 hours post ketamine; the insula and dACC response decreased 7 days post ketamine (60). Additionally, the substantia nigra/ventral tegmental area appeared more active for negative than positive picture ratings pretreatment, a pattern that reversed 7 days following ketamine (60). Because the amygdala, insula, and dACC are often found to be overreactive when processing negatively valanced stimuli in MDD (64,65), these findings suggest that ketamine may normalize this particular hyperactive signature of MDD.

Using an attentional bias dot probe task with emotional face stimuli to investigate attentional bias pre- to post-ketamine, MDD participants showed a reversal of PFC and dACC activity for happy and angry trials 1 to 3 days post ketamine versus placebo (66). Reductions in depressive symptoms (38) associated with decreased and increased activation for angry and happy trials, respectively, in the left parahippocampus and amygdala, bilateral ACC, precuneus, and left PFC. Using an implicit and explicit facial recognition task, the same group showed reduced activity post ketamine in the frontal, temporal, and precuneus regions in patients that normalized toward healthy control subjects (67). Further, greater differences in left temporal and bilateral precuneus activation were observed between explicit and implicit conditions post ketamine in MDD. Conversely, a separate study found a significant increase in neural response to positively valanced faces (happy>neutral) in the right caudate that was linked with improved depressive symptoms (38) 24 hours following single-dose ketamine (68). Another study used an emotional n-back task with verbal stimuli and found significant associations between lower baseline dorsomedial PFC activity and improvements in cognitive but not depressive symptoms (69). Only one study has investigated emotion recognition following serial ketamine treatment (70). Here, both patients receiving ketamine or electroconvulsive therapy showed a decrease in amygdala response while processing positive and negative face stimuli. Changes in inferior parietal activity correlated with overall symptom improvement, and the BOLD signal change in frontal regions correlated with anxiety and anhedonia.

Two studies have correlated change in BOLD signal during ketamine infusion with longer-term clinical response. Results showed significant associations with increased BOLD response in the ACC (71) and in the right insula and left postcentral gyrus (72) with improved depressive outcomes.

#### Summary.

**Figure 3.**
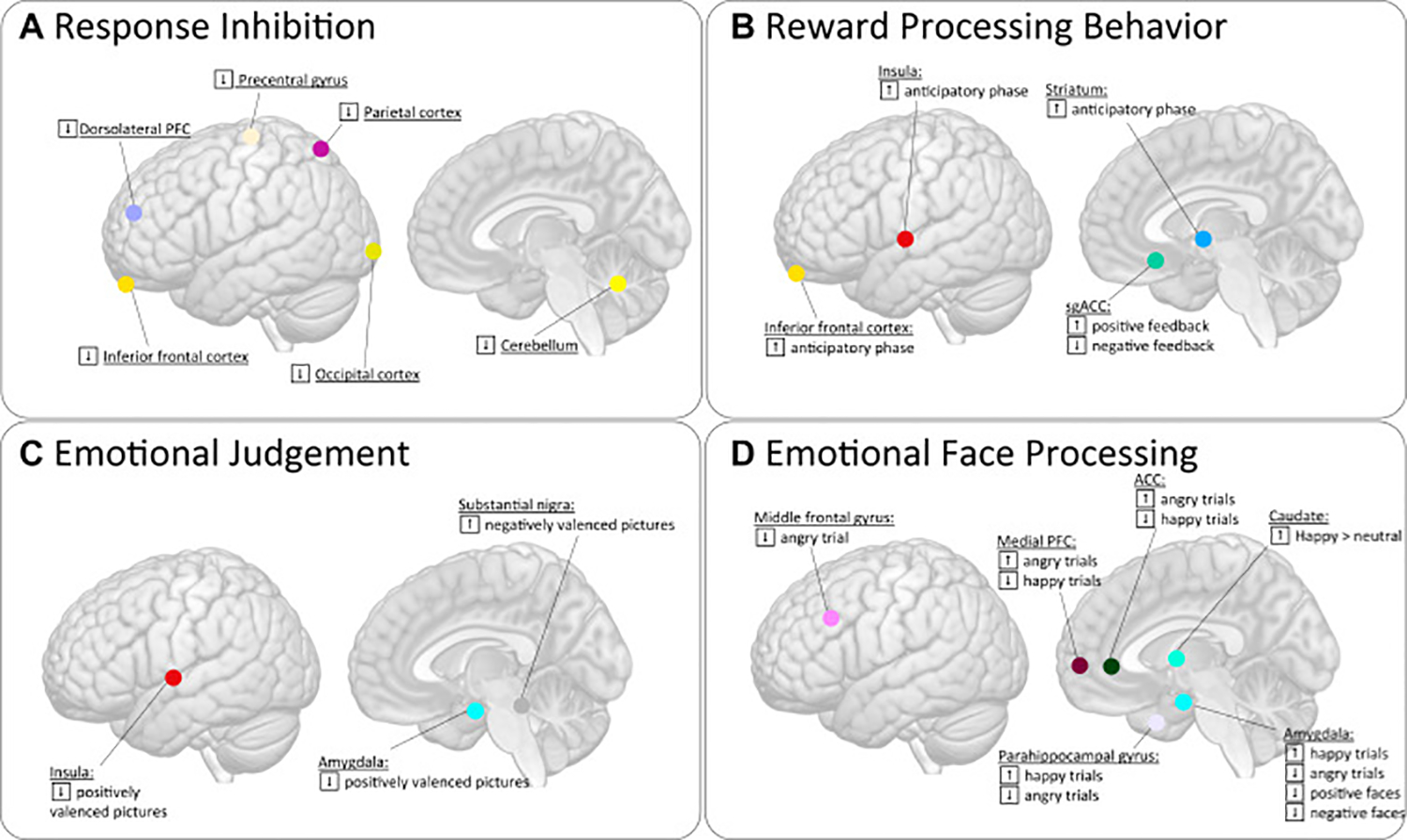
This illustration summarizes results from functional imaging studies using **(A)** response inhibition, **(B)** reward processing, **(C)** emotional judgment, and **(D)** emotional face recognition tasks to investigate neural changes in functional systems associated with ketamine treatment in major depressive disorder. Increases and decreases of brain activity observed are displayed by task. Table S2 indicates corresponding references to changes in brain activity. ACC, anterior cingulate cortex; PFC, prefrontal cortex; sgACC, subgenual anterior cingulate cortex.

To date, fMRI studies of ketamine response have focused on probing functional systems of reward, emotion, and cognitive control. Considering the differences in tasks and neural targets, only partial consensus exists among studies (Figure 3). However, all found that ketamine perturbs components of MDD-relevant neural circuits. Notably, regional changes in brain activation during response inhibition (PFC, parietal, and cerebellar regions) associated with improved mood (55,56), while regional BOLD response changes (sgACC) during reward processing associated with anhedonia (59). Tasks assessing emotional response showed that changes in neural signal in the limbic regions, the caudate, PFC, and parietal cortex were related to improved clinical response (66,68,70) or to anhedonia (70).

## POSITRON EMISSION TOMOGRAPHY

Positron emission tomography (PET) measures metabolic or biochemical neural processes using radioactive tracers (73). All identified PET publications meeting inclusion criteria studied single-dose ketamine infusion (Table 3).

Most PET studies evaluating ketamine response at least 24 hours posttreatment in MDD used [^18^F]-fluorodeoxyglucose PET to measure glucose metabolism, a proxy for estimating glutamatergic neurotransmission (73). These findings suggest that increased glucose metabolism in the dACC post ketamine is linked with improved anhedonia (35) in both unipolar (74) and bipolar (75) depression and with improved depressive symptoms (37) in treatment-resistant depression (76). The dACC functions to integrate emotional and cognitive processes and is likewise implicated in rsfMRI and task-based fMRI ketamine studies (see previous sections). Post ketamine improvements in anhedonia have also been associated with increased [^18^F]-fluorodeoxyglucose signal in the hippocampus (74), supplementary motor area (76), and cerebellum (75), regions also implicated in fMRI studies. Increased glucose metabolism in the hippocampus and amygdala has been specifically observed in patients with bipolar depression with higher baseline levels of SHANK3, a protein involved in glutamatergic neurotransmission, following single ketamine infusion. However, findings did not associate with changes in depressive symptoms >24 hours posttreatment (38,77). Using a selective serotonin (5-HT_1B_) receptor radioligand to investigate 5-HT_1B_ receptor binding in selective serotonin reuptake inhibitor–resistant depression, one study found a 17% increase in nondisplaceable binding potential (BPND) to the 5-HT_1B_ receptor in the hippocampus 1 to 3 days following ketamine infusion (78). Additionally, baseline BPND in both the ventral striatum and the dorsal brainstem was inversely correlated with changes in depressive symptoms (38); however, changes in BPND were not correlated with antidepressant effects.

### Summary

The PET literature suggests that regional changes in glucose metabolism post ketamine are associated with improvements in overall depressive symptoms (dACC) (74–76) and anhedonia (dACC, hippocampus, supplementary motor area, and cerebellum) (74–76). These changes in neurofunction and circuitry suggest that ketamine’s therapeutic mechanism may be at least partially attributed to downstream effects on glutamatergic signaling that may be attributable to synaptic and dendritic remodeling reported in animal studies (79). Observed increases in hippocampal BPND (78) suggest that changes in 5-HT_1B_ receptor density may contribute to ketamine’s therapeutic effects, as has been observed in conjunction with antidepressive properties of ketamine in preclinical models (80).

## ARTERIAL SPIN LABELING

Arterial spin labeling (ASL) (perfusion) MRI measures cerebral blood flow (CBF) using magnetically labeled arterial blood without the need for any radioactive ligand. ASL CBF measurements have been validated using ^15^O-water PET (81), and because perfusion is normally coupled with metabolism, yield information is comparable to [^18^F]-fluorodeoxyglucose PET (82). Thus, by providing quantitative measures of brain hemodynamics, ASL provides information regarding neurofunction that is complementary to fMRI.

Increased CBF in the thalamus has been observed 24 hours after single IV ketamine infusion (83,84). Furthermore, change in thalamic perfusion has been associated with greater reductions in depressive symptoms, and patients with lower baseline thalamic perfusion showed larger increases in perfusion after ketamine (83). One study leveraged multiband pseudo-continuous ASL MRI to compare global and regional CBF in patients with treatment-resistant depression at baseline and 24 hours after receiving a single and 4 serial ketamine infusions (85). In concordance with the direction of prior acute-response PET results (14), post single ketamine regional CBF increased in the posterior cingulate cortex and in visual association regions. Post serial infusion therapy, regional CBF decreased in the bilateral hippocampus and right insula, normalizing toward levels of healthy control subjects. Further, acute changes in CBF in visual areas predicted improvements in anhedonia, anxiety, and overall mood following treatment (85).

### Summary

ASL findings suggest that neurophysiological changes occurring with ketamine treatment include initial engagement of the thalamus (83,84) posterior ACC, precuneus (DMN nodes), and primary and higher-order visual areas (14,85) and reflect improvements in overall mood (83,85) as well as improved anhedonia and anxiety (Table 4). Continued ketamine treatment includes the subsequent engagement of deeper limbic structures and the insula (affective networks and SN).

## STRUCTURAL MRI

Fewer studies have addressed how subanesthetic ketamine impacts brain structure, likely due to the expectation that structural plasticity occurs slowly, and most ketamine MRI investigations only include short follow-ups (Table 5).

### Subcortical Regions

Reductions of hippocampal volume are well replicated in MDD and appear to be influenced by clinical state (86–88) and other antidepressant interventions (89–91). Initial evidence suggests that single and serial ketamine treatment may also impact hippocampal volume. Increased right gross hippocampal volumes have been observed 24 hours post serial infusion (92). At the hippocampal subfields level, ketamine-induced increases in the right CA4 head/molecular layer (ML) and left CA4 body, and dentate gyrus (DG)/granule cell (GC)/ML are reported; increases in the left CA1 body, CA4 body, bilateral GC-ML-DG, and right ML head occurred in responders only (93). An independent investigation observed increased left gross hippocampal volumes in ketamine remitters but not in nonremitters after single-dose ketamine (94).

Previous studies suggest that pretreatment hippocampal volume may influence subsequent response to pharmaco-therapy in MDD (95,96). One study (30) found that smaller pretreatment right gross hippocampal volume associated with greater reductions in depressive symptoms (38) following a 96-hour infusion. After standard single infusion treatments, one study reported that larger left pretreatment hippocampal volume was associated with greater antidepressant response (38,97); however, another study found no associations between pretreatment hippocampal volume and antidepressant response (98). Larger pretreatment left anterior subiculum volumes in serial ketamine responders have been observed (92). Pretreatment volumes of the right thalamus have also been associated with clinical response (38) following 6 serial ketamine infusions (93); however, these results were not found after single ketamine treatment in an independent study (98).

Though evidence is limited, ketamine may also affect the structure of other subcortical structures. Following serial ketamine, increased left amygdalar volume was correlated with antidepressant response (38,92). Another study observed decreased left nucleus accumbens volume following treatment in remitters only (94). However, increased nucleus accumbens, bilateral putamen, thalamus, caudate, and periaqueductal gray matter volume was detected following 6-week oral (racemic) ketamine treatment in voxel-based analyses (99). Finally, decreases in midbrain volumes 24 hours after single ketamine infusion have been reported (100).

### Cortical Regions

Decreased volumes of the left lateral OFC and an increase in the right precentral gyrus, right opercular inferior frontal gyrus, right operculum, right insula, and right postcentral gyrus have been reported 24 hours after ketamine (100). In contrast, an independent report found no significant changes in cortical volume post ketamine (99). One report showed a positive association between baseline ACC volume and change in depressive symptoms (37,38) 24 hours post single infusion (101).

### Summary

Existing data suggest that subanesthetic ketamine may lead to hippocampal structural plasticity (93,97), despite mixed laterality of reported effects, and may also relate to antidepressant response. Notably, preclinical models have shown that ketamine induces neurotrophic processes, such as increased expression of brain-derived neurotrophic factor, and increased hippocampal spine density (102) that may contribute to observed changes in hippocampal macrostructure (92–94). Other subcortical regions are also implicated in isolated studies (94,99), and effects may be more pronounced after serial ketamine treatment (93,99). Some data suggest that ketamine leads to changes in higher cortical areas, but studies are limited (100,101). Existing data also suggest that pretreatment hippocampal (sub)structure (30,93,97) and thalamic (93) volumes might associate with subsequent clinical response.

## DIFFUSION-WEIGHTED MRI

Diffusion-weighted MRI tracks and quantifies water diffusion in brain tissue to evaluate white matter microstructure and other tissue properties (103). In a preliminary study, patients who responded to single IV ketamine had significantly higher fractional anisotropy (FA) and lower radial diffusivity in the cingulum and forceps minor at baseline compared with non-responders, suggesting that an individual’s predisposition for ketamine response may be influenced by regional white matter microstructure (104). When investigating changes in diffusion metrics 4 hours after single ketamine infusion, increases in FA in the forceps minor and bilateral uncinate fasciculus negatively correlated with symptom improvement (37) 24 hours postinfusion (105). Further, greater pre single infusion FA in the cingulum (hippocampal portion) and left superior longitudinal fasciculus associated with improved depressive symptoms (37) 24 hours posttreatment (105). In supplementary analysis, lower baseline FA in tracts connecting the right amygdala and sgACC was associated with improved clinical response (38,106).

### Summary

Together, diffusion-weighted MRI studies in MDD, though few, suggest that ketamine may have a unique effect on white matter microstructure and connectivity (Table 6). However, more elaborate models of diffusion (107,108) have not yet been investigated within the context of ketamine treatment and may reveal other measurable changes in regional white matter tissue properties.

## MAGNETIC RESONANCE SPECTROSCOPY

Magnetic resonance spectroscopy may reveal changes in glutamate signaling or other aspects of cellular integrity and metabolism linked with or predictive of ketamine’s antidepressant effects. However, none of the 4 published studies using single-voxel proton (^1^H) MRS to investigate downstream changes in brain metabolites ≥1-day post single IV ketamine infusion detected significant changes in brain metabolites or associations with ketamine response in MDD (109–112) (Table 7).

## CONCLUSIONS

A growing number of neuroimaging studies have investigated brain systems–level effects of subanesthetic ketamine treatment in MDD. Although results are difficult to reconcile given the differing study designs and analyses, recurring patterns of neurofunctional plasticity are evident. The fMRI literature consistently suggests that ketamine impacts depression-relevant functional brain networks and brain activity, most frequently implicating networks encompassing prefrontal, limbic, and striatal regions. Several studies reported increased frontostriatal connectivity (42,47,51) and decreased interlimbic connectivity (30,40), often associated with improved depressive symptoms and normalizing toward values in healthy control subjects. This suggests that part of ketamine’s therapeutic mechanism may be driven by its ability to regain cognitive control over emotional activity. PET and perfusion ASL studies have also identified networks and regions that may serve as biomarkers of ketamine response, including the ACC (74–76), striatal (78) and cerebellar (75) circuitry, and visual regions (14,85). These summarized changes in functional brain circuitry may be driven by rapidly occurring synapto- and dendrogenesis observed in signaling pathways downstream to NMDA receptor– or AMPA-mediated actions in preclinical models of ketamine treatment (113,114).

Given the small sample sizes, heterogeneity of depression, and variability in imaging analysis methods, there is a need for systematic replication of prior results, which should be a goal of future studies. Though participants serve as their own control subjects in longitudinal studies, differences concerning whether concurrent antidepressant medication is allowed and stable could potentially influence results. Thus, more refined investigation of the effects of simultaneous antidepressant treatment may assist in deciphering underlying response mechanisms to benefit treatment approaches. While considering the limitations of the current literature (Table 8), sample demographics and neuroimaging parameters (Tables S3–S16) and study designs (Tables 1–7) are available for readers to evaluate the potential impact of each study, contextualize findings, and inform the design of future studies.

These reviewed studies have provided important leads regarding how ketamine modulates structural and functional brain systems to elicit antidepressant effects and have pointed to neurobiological features that appear to influence subsequent ketamine response in MDD. However, there is a lack of replication, and data-driven cross-validation that may more directly link macrostructural properties or changes to ketamine’s underlying antidepressant mechanisms, which are required before this work can meaningfully impact clinical decision making and patient outcomes.

## Supplementary Material

Supplemental Material

## Figures and Tables

**Figure 1.**
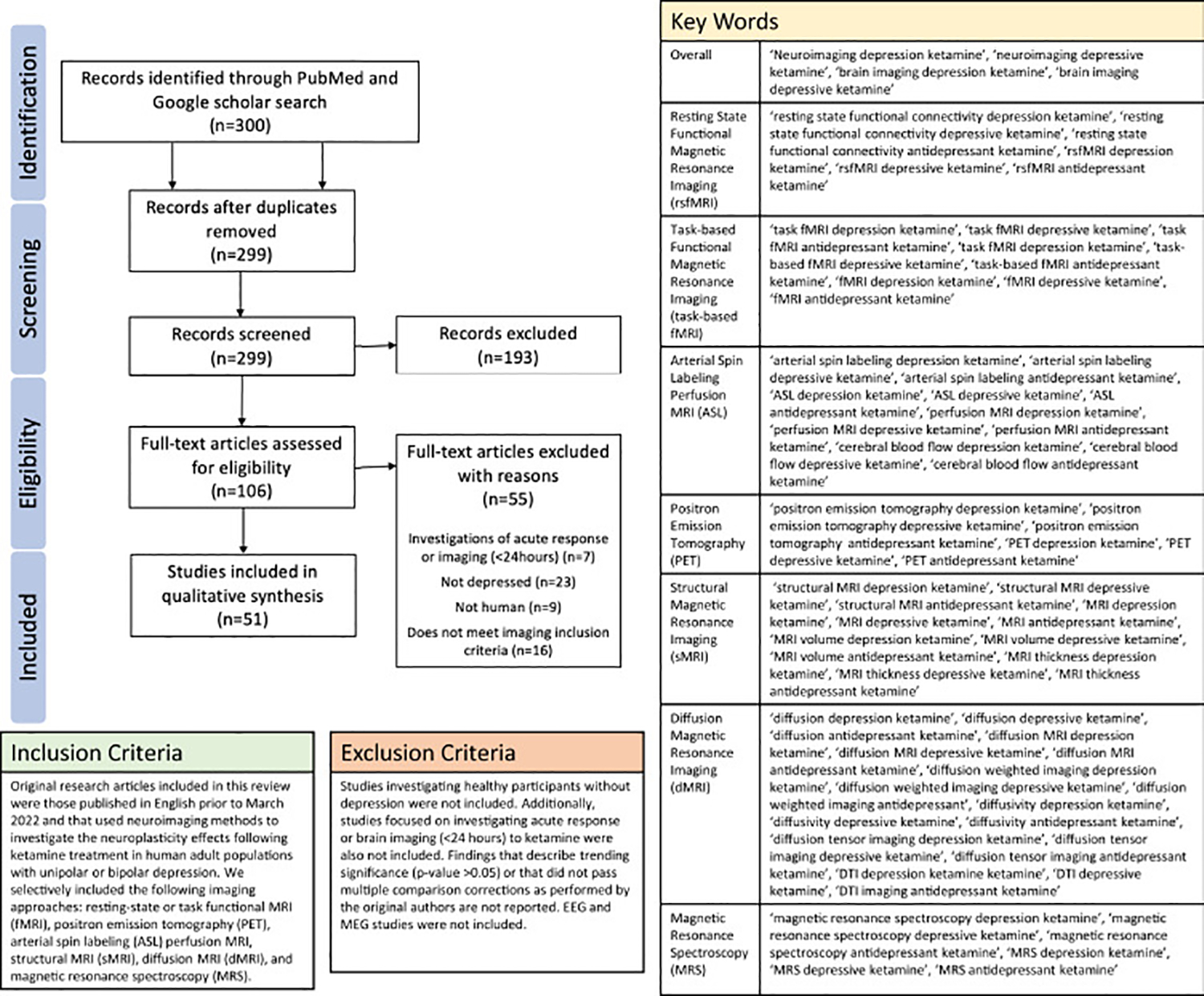
Original research articles were identified and evaluated according to the PRISMA (Preferred Reporting Items for Systematic Reviews and Meta-Analyses) review method. Literature searches were performed in the PubMed and Google Scholar databases using key words (right) and the inclusion/exclusion criteria (bottom) provided. For more details regarding methods, see the Supplement. EEG, electroencephalography; MEG, magnetoencephalography; MRI, magnetic resonance imaging.

**Table 1.** Resting-State fMRI Studies of Ketamine Treatment in Depressed Populations

Sample	Other Medication	Ket Treatment	Data Collection	Depression Assessment	Summary of Findings
Abdallah *et al*., 2017 (49); NCT00768430					
*n*^a^: 22 Unipolar TRD***, 47 HC^§^	NO: antidepressant- and antipsychotic-free ≥ 1 wk prior to treatment	Cohort A^++^: Single IV ket, 0.5 mg/kg over 40 min Placebo: 0.45 mg/kg midazolam over 40 min	Cohort A neuroimaging: Baseline 24 h postinfusion	MADRS, QIDS, Brief Psychiatric Rating Scale, Clinician-Administered Dissociative State	(+) GBCr post ket in bilateral dmPFC and right dlPFC for TRD, normalizing to HC No significant change in GBCr detected in placebo group
		Cohort B^++^: Single IV ket, 0.23 mg/kg in 2 min followed by 0.58 mg/kg over 70 min Placebo: oral placebo Saline IV	Cohort B neuroimaging: During infusion	Scale	
Abdallah *et al*., 2017 (47); NCT00548964, NCT00768430, NCT01880593					
*n*: 18 Unipolar TRD**, 25 HC	NO: antidepressant- and antipsychotic-free ≥ 1 wk prior to treatment; benzodiazepines witheld at day of each scan	Single IV infusion 0.5 mg/kg ket over 40 min^+^	Neuroimaging: Baseline 24 h postinfusion Clinical assessment: Baseline 24 h postinfusion	MADRS: response was defined as ≥ 50% reduction in MADRS	(+) GBCr in right lateral PFC after treatment for all TRD participants (−) GBCr in left cerebellum after treatment for all TRD participants (+) Change in GBCr (post-pre ket) in right lateral PFC and left anterior insula in Rs (+) GBCr post ket in bilateral caudate, bilateral lateral PFC, and left middle temporal lobe of Rs (−) FC within PFC after ket (+) FC between PFC and other brain regions after ket
Abdallah *et al*., 2018 (48); NCT01046630					
*N*: 58 Unipolar MDD	NO	Single IV infusion 0.5 mg/kg of ket, 100 mg of lanicemine, or saline placebo over 60 min^++^	Neuroimaging and clinical assessment: Baseline During Infusion 24 h postinfusion	BDI	(+) PFC GBCr in ket during infusion and at 24 h posttreatment compared with baseline (+) GBCr in dlPFC, dmPFC, and fmPFC during ket infusion (+) GBCr in dorsolateral and dorsomedial PFC at 24 h posttreatment Vertexwise analysis showed depression improvement positively correlated with GBCr in dPFC during infusion and 24 h posttreatment but negatively correlated with GBCr in ventral PFC during infusion
Evans *et al*., 2018 (31); NCT00088699					
*n*: 33 Unipolar TRD*, 25 HC^§^	NO: medication-free ≥ 2 wk prior to treatment	Single IV infusion 0.5 mg/kg ket or saline placebo during first session followed by alternative treatment 2 wk later^+++^	Neuroimaging: Baseline 2 and 10 days postinfusion Clinical assessment: Baseline 40, 80, 120, and 230 min postinfusion on days 1, 2, 3, 7, 10, and 11	MADRS	(+) RSFC between bilateral insula, middle frontal gyrus, postcentral gyrus, and occipital gyrus and DMN 2 days after ket infusion compared with placebo infusion that was no longer evident at day 10 RSFC between insula (strongest in right posterior insula) and DMN normalized for MDD 2 days after ket—no significant difference between TRD and HC—that returned to baseline at day 10
Chen et *al*., 2019 (33); UMIN000016985					
*N*: 48 Unipolar TRD**	YES: permitted to remain on antidepressants	Single IV infusion 0.5 mg/kg ket, 0.2 mg/kg ket, or saline placebo over 40 min^+++^	Neuroimaging and clinical assessment: Baseline 48 h postinfusion	MADRS	(−) RSFC between left and right dACC after ket, associated with reduction in suicidal ideation (−) in RSFC between dlPFC and right frontal pole in 0.5-mg/kg group (+) RSFC between right dlPFC and left superior parietal lobe associated with reduced suicidal ideation in 0.2-mg/kg group (+) RSFC between right dACC and right anterior middle temporal gyrus in 0.2-mg/kg group
Gärtner et *al*., 2019 (39); NCT02099630, NCT03609190					
*N*^b^: 24 Unipolar TRD**	YES: prior antidepressant medication remained unchanged	Site A: single IV infusion 0.5 mg/kg racemic ket^+^	Neuroimaging and clinical assessment: Baseline 24 h postinfusion	MADRS, HDRS	(+) RSFC between sgACC and 4 regions (SMA, dlPFC, anterior PFC, and OFC) associated with symptom reduction ↓ Whole brain connectivity at baseline associated with (−) depressive symptoms 24 h after ket
		Site B: single IV infusion 0.25 mg/kg S-ket^+^			
Chen *et al*., 2020 (41); UMIN000016985					
*n*: 48 Unipolar TRD**	YES: concomitant stable antidepressant treatment ≥ 2 wk prior	Single IV infusion at 0.5 mg/kg or 0.2 mg/kg ket or saline placebo over 40 min^+++^	Neuroimaging: Baseline Clinical assessment: Baseline 40, 80, 120, and 240 min postinfusion 2, 3, 4, 5, 6, 7, and 14 days postinfusion	HDRS	↓ Baseline RSFC between bilateral superior frontal cortex and striatum associated with (−) depressive symptoms after 0.2-mg/kg ket infusion
Kraus *et al*., 2020 (50); NCT00088699					
*n*: 28 Unipolar TRD*, 22 HC^§^	NO: medication-free ≥ 2 wk prior to treatment	Single IV infusion 0.5 mg/kg ket or saline placebo during first session followed by alternative treatment 2 wk later^+++^	Neuroimaging: Baseline 2 and 3 days postinfusion Baseline Clinical assessment: Before, during, and after each infusion	MADRS, BDI, HARS	Could not replicate (+) connectivity observed in Abdallah *et al*. (47) after ket treatment
Sahib *et al*., 2020 (46); NCT02165449					
*n*: 61 Unipolar TRD**, 40 HC	YES: monoaminergic antidepressants allowed if stable ≥ 6 wk prior to treatment; no benzodiazapine ≤ 72 h prior to treatment	4 serial IV infusions of 0.5 mg/kg ket over 40 min^+^	Neuroimaging and clinical assessment: Baseline 24 h post first infusion 24–72 h post fourth infusion	HDRS-17; Rms: posttreatment HDRS ≤ 7	(+) RSFC within SMN after ket, normalizing toward HC (−) RSFC between ventral node and visual node after ket, normalizing toward HC. ↑ RSFC between visual cortex and DMN at baseline that (−) with each infusion. (−) RSFC between cerebellum and SN between first and fourth ket treatment, normalizing toward HC (−) RSFC between cerebellum and striatum for Rm, normalizing toward HC Opposite effect in NRm pre- and posttreatment change in whole brain FC significantly different between Rs and NRs
Zhuo *et al*., 2020 (51)					
*N*: 38 Bipolar TRD**	YES: remained on mood stabilizer or antipsychotic medication if clinically stable	Serial IV infusion of 0.5 mg/kg ket over 40–50 min on days 2, 4, 6, 8, 10, 12, 15, 18, and 20 of study^+^	Neuroimaging: Baseline Days 2, 7, 14, and 21 after beginning treatment	HDRS-17	Changes in GBCd after first day of ket and peaked at day 7. Effects mostly gone by end of third wk. (−) GBCd in bilateral insula, right caudate nucleus, and bilateral inferior frontal gyrus (+) GBCd in bilateral postcentral gyrus, sgACC, bilateral thalamus, and cerebellum No significant associations with clinical measure
Mkrtchian *et al*., 2020 (42); NCT00088699					
*n*: 33 Unipolar TRD*, 25 HC^§^	NO: medication-free ≥ 2 wk prior to treatment	Single IV infusion of 0.5 mg/kg ket or saline placebo during first session followed by alternative treatment 2 wk later^+++^	Neuroimaging: 2 days postinfusion Clinical assessment: Baseline 40, 80, 120, and 230 min 1,2,3, 7, 10, and 11 days postinfusion	MADRS, SHAPS	(+) RSFC between VS-left dlPFC, DC–right vlPFC, DC-pgACC, and VRP-OFC in TRD but (−) in HC post ket (+) RSFC in DC–right vlPFC correlated with improved day 2 SHAPS (+) RSFC in DC-pgACC correlated with improved day 10 SHAPS
Nakamura *et al*., 2021 (40); UMIN000017529					
*n*: 14 Unipolar TRD***, 1 bipolar TRD*	NO: discontinuation of antidepressant medications	Serial IV infusions of ket over 40 min twice a wk over 2 wk (4 total) with concurrent daily oral placebo or lithium carbonate (600–800 mg) daily^++^	Neuroimaging: Baseline 6–24 h postinfusion Clinical assessment: 4 h post final infusion	MADRS, Young Mania Rating Scale; Rs: decrease in MADRS ≤ 50%	Significant cluster defining Rs and NRs between the amygdala and sgACC in the right AN (−) RSFC between amygdala and sc/sgACC in the right hemisphere at baseline associated with (+) depressive symptoms (MADRS) RSFC between amygdala and scACC/sgACC in the right hemisphere Rs > NRs both at baseline and follow-up
Siegel *et al*., 2021 (30); NCT01179009					
*n*: 23 Unipolar TRD**, 27 HC	YES: SSRI and SNRI allowed if constant for ≥ 6 wk prior to infusion	Continuous 96-h IV infusion of ket started at 0.15 mg/kg/h at 10 am on day 1 and titrated to tolerance twice daily to target rate of 0.6 mg/kg/h^+^	Neuroimaging: Baseline 2 wk postinfusion Clinical assessment: 2, 4, 6, and 8 wk postinfusion	MADRS	(−) RSFC within DMN 2 wk after ket (−) RSFC between sgACC and DMN 2 wk after ket across hemispheres (+) RSFC between sgACC and bilateral cACC and bilateral anterior insula (−) RSFC within limbic system, especially with anterior thalamus (+) RSFC between limbic regions and cortical areas, especially cingulo-opercular network
Rivas-Grajales *et al*., 2021 (44); NCT00768430, NCT01880593					
*N*: 35 Unipolar TRD**	NO: antidepressant-free ≥ 1 wk prior to treatment	Single IV infusion of 0.5 mg/kg ket over 40 min^+^	Neuroimaging and clinical assessment: Baseline 24 h postinfusion	MADRS, QIDS-SR	(+) RSFC between right habenula and right frontal pole of dlPFC associated with (−) MADRS scores (+) RSFC between right habenula and right occipital pole, right temporal pole, right parahippocampal gyrus, and left lateral occipital cortex associated with (−) QIDS-SR
Vasavada *et al*., 2021 (34); NCT02165449					
*n*: 44 Unipolar TRD**, 50 HC	YES: monoaminergic antidepressants allowed if stable ≥ 6 wk prior to treatment; no benzodiazapine ≤ 72 h prior to treatment	4 serial IV infusions of 0.5 mg/kg ket over 40 min^+^	Neuroimaging and clinical assessment: Baseline 24 h post first infusion 24–72 h post fourth infusion	HDRS-17, SHAPS, DASS, BIS	(+) RSFC between right amygdala and right CEN normalizing toward HC with each infusion (−) RSFC between left amygdala and SN with each infusion correlated with improved BISS Greater negative connectivity between right hippocampus and left CEN correlated with improved SHAPS
Zhang *et al*., 2020 (32)					
*N*: 36 Unipolar TRD**	Not reported	6 serial IV infusions of 0.5 mg/kg over 40 min followed by propofol-ECT treatment 24 h later^+^	Clinical assessment: Baseline First, third, seventh, 10th, and 14th days after first treatment Neuroimaging: Baseline Seventh and 14th days of treatment	HDRS	(−) GBCd in medial prefrontal lobe, sgACC, posterior cingulate cortex, thalamus, hippocampus, and orbitofrontal lobe on day 7 (−) FC within DMN through third day

↑ indicates higher, ↓ indicates lower, (+) indicates increase, and (−) indicates decrease.

AN, affective network; BDI, Beck Depression Inventory; BIS, Behavioral Inhibition System Scale; cACC, caudal anterior cingulate cortex; CEN, central executive network; dACC, dorsal anterior cingulate cortex; DASS, Depression Anxiety and Stress Scale; DC, dorsal cingulate; dlPFC, dorsolateral prefrontal cortex; DMN, default mode network; dmPFC, dorsomedial prefrontal cortex; dPFC, dorsal prefrontal cortex; ECT, electroconvulsive therapy; FC, functional connectivity; fmPFC, frontomedial prefrontal cortex; fMRI, functional magnetic resonance imaging; GBCd, global functional connectivity density; GBCr, global brain connectivity regression; HARS, Hamilton Anxiety Rating Scale; HC, healthy control; HDRS, Hamilton Depression Rating Scale; IV, intravenous; ket, ketamine; MADRS, Montgomery–Åsberg Depression Rating Scale; MDD, major depressive disorder; NR, nonresponder; NRm, nonremitter; OFC, orbitofrontal cortex; pgACC, pregenual anterior cingulate cortex; PFC, prefrontal cortex; QIDS, Quick Inventory of Depressive Symptomatology; QIDS-SR, Quick Inventory of Depressive Symptomatology–Self-Report; R, responder; Rm, remitter; RSFC, resting-state functional connectivity; scACC, subcallosal anterior cingulate cortex; sgACC, subgenual anterior cingulate cortex; SHAPS, Snaith–Hamilton Pleasure Scale; SMA, supplementary motor area; SMN, somatomotor network; SN, salience network; SNRI, serotonin and norepinephrine reuptake inhibitor; SSRI, selective serotonin reuptake inhibitor; TRD, treatment-resistant depression; vlPFC, ventrolateral prefrontal cortex; VRP, ventral rostral putamen; VS, ventral striatum.

a Two studies were pooled. Cohort A was conducted at one site in Houston as an add-on to a ketamine clinical trial. Cohort B was conducted at a different site in New Haven, Connecticut, to investigate modulation of ketamine effects by lamotrigine and only included HC subjects.

b Two different scanners were used to collect resting-state functional magnetic resonance imaging data. Scanning site used as a covariate in the analysis. Post hoc analysis found similar results when testing sites individually.

* ≥1 failed treatment.

** ≥2 failed treatment.

*** ≥3 failed treatment.

§ HC received ketamine.

+ Open label.

++ Randomized placebo.

+++ Randomized crossover.

**Table 2.** Task-Based fMRI Studies of Ketamine Treatment in Depressed Populations

Sample	Other Medication	Ket Treatment	Data Collection	Depression Assessment	Summary of Findings
Sahib *et al*., 2020 (55); NCT02165449					
*n*: 47 Unipolar TRD**, 32 HC	YES: monoaminergic antidepressants allowed if stable ≥ 6 wk prior to treatment; no benzodiazapine ≤ 72 h prior to treatment	4 serial IV infusions 0.5 mg/kg ket over 40 min^+^	Neuroimaging and clinical assessment: Baseline 24 h post first infusion 24–72 h post fourth infusion	HDRS-17; Rms: end of treatment HDRS ≤ 7	Conditioned approach response inhibition: (−) Activation between baseline and fourth infusion in inferior frontal cortex and dlPFC along superior and inferior parietal regions and right cerebellum (−) Activation in visual cortex and superior parietal regions of left hemisphere after fourth infusion (−) BOLD activity for DMN, FPN, DAN, and SN in right hemisphere following ket (−) NoGo>Go activity in bilateral precentral gyrus for NRms after serial ket (+) NoGo>Go activity in bilateral precentral gyrus for Rms after serial ket Average baseline contrast values in bilateral precentral gyrus show negative correlation with % change in HDRS score after serial treatment (−) Contrast values in bilateral precentral gyrus 24 h post ket significantly associated with serial HDRS improvement
Loureiro *et al*., 2021 (56); NCT02165449					
*n*^a^: 46 Unipolar TRD**, 32 HC	YES: monoaminergic antidepressants allowed if stable ≥ 6 wk prior to treatment; no benzodiazapine ≤ 72 h prior to treatment	4 serial IV infusions 0.5 mg/kg ket over 40 min^+^	Neuroimaging and clinical assessment: Baseline 24 h post first infusion 24–72 h post fourth infusion	HDRS-17, QIDS, DASS	Conditioned approach response inhibition: (−) Connectivity between cerebellum and FPN and the SMN in Rms only Baseline connectivity between cerebellum and FPN and cerebellum and SN significantly correlated with ket related % change in QIDS for both Rms and NRms
Murrough *et al*., 2015 (68); NCT00548964, NCT00768430, NCT01880593					
*n*: 18 Unipolar TRD**, 20 HC	NO: antidepressant medication–free ≥ 1 wk prior to treatment	Single IV infusion of 0.5 mg/kg ket over 40 min^+^	Neuroimaging and clinical assessment: Baseline 24 h postinfusion	MADRS	Facial emotion perception task: (+) Neural response to positive emotions (happy 100% > neutral) following ket centered in the right caudate but not correlated with pre-ket depressive symptoms (+) Post-ket right caudate connectivity associated with improved MADRS score
Sterpenich *et al*., 2019 (60); NCT01135758					
*N*: 10 Unipolar TRD**	YES: stable medications ≥ 6 wk prior to treatment	Single bolus IV infusion 0.5 mg/kg ket over 1 minute^+^	Neuroimaging: Baseline 1 day postinfusion Clinical assessment: Baseline 40, 80, 110, and 230 min postinfusion	MADRS, HDRS-21, BDI-II	Reward task adapted from monetary inventive delay task: (+) Changes in insula and OFC during anticipatory phase of reward task 1 day after ket compared with baseline and in VS and OFC 7 days after ket compared with baseline (more activated for positively cued trials) (+) Activation of VS and OFC in response to winning vs. losing when comparing day 7 with baseline (+) More active substantia nigra/ventral tegmental area when winning than losing 1 day after ket and 7 days after ket compared with baseline
					Emotional judgment task: Patient reaction time faster after ket (baseline vs. day 1 and day 7) but no effect of emotion (−) Activation of amygdala and insula response to negative pictures 1 day after ket (−) Activation of insula and dACC 7 days after ket in response to negative pictures (+) Medial substantia nigra/ventral tegmental area more active for negative than positive pictures at baseline and then became (+) active for positive compared with negative pictures at day 7
Morris *et al*., 2020 (59)					
*n*: Study 1: 28 Unipolar MDD, 20 HC	NO: antidepressant and other medication free at time of scan	Single IV infusion 0.5 mg/kg ket over 40 min^+^	Neuroimaging: Baseline 5 days postinfusion	MADRS, TEPS, STICSA	Reward incentive flanker task: (−) sgACC activation to positive feedback with ket but not to negative feedback
Study 2: 16 Unipolar TRD**					Higher pre-ket sgACC activation to positive feedback associated with better improvements in anhedonia after ket
Reed *et al*., 2018 (66); NCT00088699					
*n*: 33 Unipolar TRD*, 26 HC^§^	NO: medication-free ≥ 2 wk prior to treatment	Single IV infusion 0.5 mg/kg ket over 50 min or saline placebo followed by alternative treatment 2 wk later^+++^	Neuroimaging: Baseline Immediately after infusion 1–3 days postinfusion Clinical assessment: Baseline 40, 80, 120, and 240 min postinfusion 1, 2, 3, 7, and 10 days postinfusion	MADRS	Dot probe task with emotional face stimuli: (+) Activation post ket compared with postplacebo in left middle occipital gyrus across groups (−) Activation post ket compared with postplacebo in left temporal and inferior frontal cortices across groups (−) Activation post ket vs. postplacebo in patients with MDD in right frontal cortex, dACC, and left inferior occipital gyrus Medial prefrontal and anterior cingulate cluster showed deactivation to angry trials and activation to happy trials in participants with MDD post placebo and reversed after ket % change in MADRS significantly associated with magnitude of activation (positive post ket and negative post placebo) (−) MADRS score associated with (−) activation to angry trials and greater activation to happy trials in the left parahippocampal gyrus and amygdala, bilateral cingulate gyri, precuneus, and left medial and middle frontal gyri
Reed *et al*., 2019 (67); NCT00088699					
*n*: 33 Unipolar TRD*, 24 HC^§^	NO: medication-free ≥ 2 wk prior to treatment	Single IV infusion of 0.5 mg/kg over 50 min and single IV of saline solution placebo 2 wk apart^+++^	Neuroimaging: Baseline Immediately after infusion 1 −3 days post infusion Clinical assessment: Baseline 40, 80, 120, and 240 min postinfusion 1, 2, 3, 7, and 10 days postinfusion	MADRS	Facial recognition task: (−) Activation post ket in bilateral frontal, temporal, precuneus, and posterior cingulate regions in MDD, normalizing toward HC when compared with placebo Greater difference in activity pattern in the left temporal gyri and bilateral precuneus/posterior cingulate between explicit and implicit processing conditions in participants with MDD after ket
Loureiro *et al*., 2020 (70); NCT02165449					
*n*: 27 Unipolar TRD**, 31 HC	YES: monoaminergic antidepressants allowed if stable ≥ 6 wk prior to treatment; no benzodiazapine ≤ 72 h prior to treatment	4 serial IV infusions of 0.5 mg/kg ket over 40 min^+^	Neuroimaging and clinical assessment: Baseline 24 h post first infusion 24–72 h post fourth infusion	HDRS, SHAPS, DASS	Emotional Faces Task: Posttreatment change in fearful objects contrast correlated with % DASS and % SHAPS change in right amygdala
Downey *et al*., 2016 (71); NCT01046630					
*N*: 56 Unipolar MDD	NO	Single IV 0.5 mg/kg ket, 100 mg lanicemine, or saline placebo over 60 min^++^	Neuroimaging: Baseline During infusion Clinical assessment: Baseline Immediately postinfusion 4 and 24 h postinfusion	BDI, MADRS	PHMRI: (+) BOLD response by both drugs in ACC predicted symptomatic improvement 24 h and 1 wk following infusion but no antidepressant effect when compared with placebo
McMillan *et al*., 2020 (72); ACTRN12615000573550					
*N*: 26 Unipolar MDD	YES: stable medications ≥ 4 wk prior	Single IV 0.25 mg/kg bolus ket followed by 0.25 mg/kg over 45 min or placebo separated by 3-wk period^+++^	Neuroimaging: During infusion Clinical assessment: Baseline 3 h, 1 day, 1 wk, and 2 wk postinfusion	MADRS	PHMRI: (+) BOLD response in right insula and left postcentral gyrus during ket infusion associated with antidepressant response
Stippl *et al*., 2021 (69)					
*N*: 16 Unipolar MDD	YES: patients permitted to remain on psychopharmacological medication	Single IV 0.25 mg/kg S-ketamine or 0.5 mg/kg racemic ket^+^	Neuroimaging: Baseline Clinical assessment: Baseline 24 h postinfusion	HDRS, BDI	Emotional working memory task: No significant association with change in depressive symptoms

(+) indicates increase and (−) indicates decrease.

ACC, anterior cingulate cortex; BOLD, blood oxygen level dependent; DAN, dorsal attention network; FPN, frontoparietal network; PHMRI, pharmacological magnetic resonance imaging; STICSA, State-Trait Inventory of Cognitive and Somatic Anxiety; TEPS, Temporal Experience of Pleasure Scale; see Table 1 for other abbreviations.

a Loureiro *et al*. (56) reported different sample sizes for each time point. T1 represents baseline, T2 represents 24 h after single infusion, and T3 represents 24 h after fourth infusion.

* ≥1 failed treatment.

** ≥2 failed treatment.

§ HC received ket.

+ Open label.

++ Randomized placebo.

+++ Randomized crossover.

**Table 3.** PET Studies of Ketamine Treatment in Depressed Populations

Sample	Other Medication	Ket Treatment	Data Collection	Depression Assessment	Summary of Findings
Esterlis et *al*., 2018 (115)					
*n*: 14 Unipolar MDD, 13 HC^§^	NO: no psychiatric medication in month prior to study	Single IV infusion initial bolus 0.23 mg/kg ket over 1 minute followed by constant infusion of 0.58 mg/kg over 1 h^+^	Neuroimaging: Baseline During infusion 24 h postinfusion Clinical assessment: 30 min and 24 h postinfusion	MADRS, BDI-II	Tracer [^11^C]ABP688: Widespread binding reductions from baseline for observed during ket and 24 h after infusion for both HC and MDD Not significantly different between diagnoses ↓ mGluR5 in hippocampus associated with (−) depression symptoms (MADRS and BDI-II)
Tiger *et al*., 2020 (78)					
*N*: 30 Unipolar TRD*	NO: ongoing medication washed out corresponding to ≥ 5 times the half-life of the drug	4 serial IV infusions of 0.5 mg/kg ket or placebo (isotonic NaCl) over 40 min given twice a wk over 2 wk^++^	Neuroimaging: Baseline 24–72 h postinfusion Clinical assessment: Baseline During PET 1,2, 3, 18, and 24 h postinfusion	MADRS, MINI, QIDS-SR, PHQ, CGIS, EQ-5D	Tracer [^11^C]AZ10419369: No significant differences in change in BPND over time between ket and placebo 16.7% ↑ BPND in hippocampus in response to first ket infusion Inverse correlation between baseline BPND in VS and ΔMADRS after first treatment in ket group Baseline BPND in DBS correlated negatively with ΔMADRS with ket ΔBPND with treatment did not correlate with antidepressant effects
Lally *et al*., 2014 (75)					
*N*: 36 Bipolar (I or II) TRD*	YES: continued mood stabilizers, no psychotropic medication or psychotherapy ≥ 2 wk prior to treatment	Single IV infusion 0.5 mg/kg ket or placebo (0.9% saline solution) followed by alternative treatment 2 wk later^+++^	Neuroimaging: 2 h postinfusion Clinical assessment: Baseline 40, 80, 120, and 230 min postinfusion 1,2, 3, 7, 10, and 14 days postinfusion	MADRS, SHAPS	Tracer FDG: ΔVS rCMRGlu after ket significantly related to % ΔSHAPS score 230 min postinfusion ΔMADRS significantly predicted ΔVS rCMRGlu but not SHAPS Whole brain analysis: association between improved SHAPS and ↑ dACC and ↑ cerebellum rCMRGlu Δ Anhedonia levels following ket not related to general changes in depressive symptoms associated with ↑ dACC, pregenual cingulate/callosal region, right dPFC, fusiform gyrus, claustrum, and putamen metabolism
Lally *et al*., 2015 (74)					
*N*: 20 TRD**	NO: drug-free for ≥ 2 prior to treatment	Single IV infusion ket over 40 min^+^	Neuroimaging: Baseline Clinical assessment: Baseline 40, 80, 120, 230 min postinfusion Daily for subsequent 28 days	MADRS, SHAPS	Tracer FDG: No association between rCMRGlu and SHAPS score at baseline Baseline metabolism did not correlate with change in anhedonia Significant negative association between ↑ dACC rCMRGlu and (−) anhedonia and after ket ↑ Glucose metabolism in cluster in right hippocampus and entorhinal cortex associated with (−) anhedonia ↓ rCMRGlu in right OFC and left inferior frontal gyrus associated with (−) anhedonia
Chen *et al*., 2018 (76)					
*N*: 24 Unipolar TRD***	YES: stable antidepressant treatment ≥ 2 wk prior to treatment	Single IV infusion of 0.5 mg/kg, 0.2 mg/kg or placebo (saline) over 40 min^+++^	Neuroimaging: Baseline Clinical assessment: Baseline 40, 80, 120, 240 min postinfusion 1 day postinfusion	HDRS-17	Tracer FDG: High-dose ket treatment showed (+) SUV in supplementary motor area and dACC compared with low-dose ket treatment ΔSUV in dACC negatively associated with HDRS-17 symptoms at day 1 [(+) ΔSUV, (−) depressive symptoms]
Ortiz *et al*., 2015 (77)					
*N*: 29 Bipolar TRD*	YES: stable mood stablizer (lithium or valproate) ≥ 4 wk at therapeutic levels	Single IV infusion 0.5 mg/kg over 40 min ^+++^	Neuroimaging: Baseline Clinical assessment: Baseline 230 min postinfusion 1, 2, 3, 7, and 10 days postinfusion	MADRS	↑ Baseline SHANK3 levels associated with (+) rMRGIu in hippocampus and amygdala ΔADRS not significantly correlated with ΔrMRGIu

↑ indicates higher, ↓ indicates lower, (+) indicates increase, (−) indicates decrease, and Δ indicates change.

BPND, nondisplaceable binding potential; CGIS, Clinical Global Impressions Scale (Severity and Improvement); DBS, dorsal brainstem; EQ-5D, EuroQol-5D; F, female; FDG, [^18^F]-fluorodeoxyglucose; M, male; mGluR5, metabotropic glutamate receptor 5; MINI, Mini-International Neuropsychiatric Interview; PET, positron emission tomography; PHQ, Patient Health Questionnaire; rCMRGlu, regional cerebral glucose metabolic rate; rMRGlu, regional glucose metabolic rate; SUV, standardized uptake value of glucose metabolism; see Table 1 for other abbreviations.

* ≥1 failed treatment.

** ≥2 failed treatment.

*** ≥3 failed treatment.

§ HC received ket.

+ Open label.

++ Randomized placebo.

+++ Randomized crossover.

**Table 4.** ASL Studies of Ketamine Treatment in Depressed Populations

Sample	Other Medication	Ket Treatment	Data Collection	Depression Assessment	Summary of Findings
Sahib *et al*., 2020 (85); NCT02165449					
*n*: 22 Unipolar TRD*, 18 HC	YES: monoaminergic antidepressants allowed if stable ≥ 6 wk prior to treatment; no benzodiazapine ≤ 72 h prior to treatment	4 serial IV infusions of 0.5 mg/kg ket over 40 min^+^	Neuroimaging and clinical assessment: Baseline 24 h post first infusion 24–72 h post fourth infusion	HDRS, SHAPS	(+) Mean CBF after first infusion that normalized/decreased after fourth infusion (+) Regional CBF after first infusion in mid and posterior cingulate and proximal association areas in paracentral lobule, cuneus, precuneus, and higher-order visual association regions like fusiform (−) Baseline CBF in fusiform associated with (+) ΔHDRS after first infusion ΔCBF in cuneus after first infusion positively correlated with change in overall mood, anhedonia, and apathy after serial treatment (−) CBF in bilateral hippocampus and right insula after serial infusion
Gartner *et al*., 2022 (83)					
*N*: 21 Unipolar MDD	YES: no restrictions to permitted medication	Single IV infusion 0.5 mg/kg racemic ket or 0.25 mg/kg S-ket over 45 min^+^	Neuroimaging and clinical assessment: Baseline 24 h postinfusion	MADRS, HDRS	(+) Thalamic perfusion 24 h after ket associated with reduced symptom severity, evident in both sites and when pooled Patients with ↓ baseline thalamus perfusion showed larger ↑ perfusion after ket ↓ Thalamic perfusion at baseline associated with more reduced depressive symptoms
Gonzalez *et al*., 2020 (84)					
*N*: 11 Unipolar TRD**	YES: stable antidepressant medication ≥ 1 month prior to treatment	Single IV infusion 0.5 mg/kg ket over 40 min^+^	Neuroimaging: Baseline 1, 6, and 24 h postinfusion	MADRS, HDRS, QIDS-SR	(+) CBF in thalamus (−) CBF lateral occipital cortex NRs showed decrease CBF in ventral basal ganglia after ket

↑ indicates higher, ↓ indicates lower, (+) indicates increase, (−) indicates decrease, and Δ indicates change.

ASL, arterial spin labeling; CBF, cerebral blood flow; see Table 1 for other abbreviations.

* ≥1 failed treatment.

** ≥2 failed treatment.

+ Open label.

**Table 5.** sMRI Studies of Ketamine Treatment in Depressed Populations

Sample	Other Medication	Ket Treatment	Data Collection	Depression Assessment	Summary of Findings
Gallay *et al*., 2021 (99); ACTRN12618001412224					
*N*: 30 MDD With Chronic Suicidality	YES: concurrent psychiatric medication reported in (116)	Once weekly dose of oral ket for 6 wk, starting at 0.5 mg/kg and titrated by 0.2–0.7 mg/kg based on tolerance with maximum dose of 3.0 mg/kg at sixth treatment^+^	Neuroimaging: Baseline End of 5-wk treatment	Beck Scale for Suicidal Ideation	VBM: (+) Bilateral gray matter in putamen, thalamus, caudate, nucleus accumbens, and periaqueductal gray after ket No cortical findings
Herrera-Melendez *et al*., 2021 (101)^a^; NCT02099630, NCT03609190					
*N*: 33 Unipolar TRD** (23 CHB, 10 UZH)	YES: no constraints on antidepressant medication	CHB: Single IV of 0.5 mg/kg racemic ket over 40 min^+^	Neuroimaging and clinical assessment: Baseline 24 h postinfusion	CHB: MADRS	VBM: ↑ GMV of bilateral rostral anterior cingulate at baseline associated with greater change in depressive symptoms
				UZH: HDRS	
		UZH: Single IV of 0.25 mg/kg S-ket over 40 min^+^		Rs: Δ Depressive symptoms^b^ > 50%	
				PRs: > 25% Δ Depressive symptoms	
Dai *et al*., 2020 (100)					
*n*: 21 Unipolar MDD (10 With Comorbid PTSD), 29 HC^§^	NO: no psychotropic or regular medication in past 2 months or history of psychiatric medication	36 participants received single IV bolus infusion of 0.23 mg/kg over 1 minute followed by constant infusion of 0.58 mg/kg over 1 h^+^	Neuroimaging: Baseline 24 h postinfusion Clinical assessment: Baseline 24 and 48 h postinfusion	HDRS-24, PTSD Checklist	TBM: (−) Left lateral OFC volume in MDD after ket (+) Left angular gyrus, left inferior parietal gyrus, left middle cingulate and paracingulate gyri, left middle occipital gyrus, left supramarginal gyrus, and left precuneus 24 h after ket in full sample including comorbid PTSD (+) Right precentral gyrus, right opercular IFG, right rolandic operculum, right insula, and right postcentral gyrus 24 h after ket in unipolar MDD group (−) Midbrain area volume in MDD group 24 h after ket
		14 participants received single IV of 0.5 mg/kg over 40 min^+^			
Zhou *et al*., 2020 (92); ChiCTR-OOC-17012239					
*N*: 44 Unipolar TRD**	YES: stable medication for ≥ 4 wk prior to treatment	6 serial IV infusions of 0.5 mg/kg over 40 min 3 times weekly for 2 wk^+^	Neuroimaging and clinical assessment: Baseline 24 h post sixth infusion	MADRS; Rs defined as ΔMADRS > 50%	FreeSurfer: (+) Right hippocampal volume 24 h after last ket treatment compared with baseline No significant associations between hippocampal volume changes and inflammatory cytokine changes
Zhou *et al*., 2020 (93); ChiCTR-OOC-17012239					
*n*: 44 Unipolar TRD**, 45 HC	YES: stable medication for ≥ 4 wk prior to treatment	6 serial IV infusions of 0.5 mg/kg over 40 min 3 times weekly for 2 wk^+^	Neuroimaging and clinical assessment: Baseline 24 h post sixth infusion	MADRS; Rs defined as ΔMADRS > 50%	FreeSurfer: Baseline Rs > NRs in left hippocampal subiculum body volume (+) Left amygdala and right hippocampus volume after ket (+) Left amygdala volume in Rs after ket (+) Left hippocampal CA4 body, left GC-ML-DG body, right CA4 head, and right ML head significantly increased after ket (+) Left CA1 body, CA4 body, left and right GC-ML-DG body, and ML head of Rs after treatment (+) Left subiculum body of NRs after treatment ↑ Pretreatment volumes in right thalamus and left subiculum head hippocampal subfield correlated with greater (−) in MADRS scores Δ Left amygdala and left DCA4 body volume negatively correlated with (−) MADRS scores after treatment
Abdallah *et al*., 2015 (97); NCT00768430					
*N*: 13 Unipolar TRD***	NO: medication-free ≥ 1 wk prior to treatment	Single IV infusion of 0.5 mg/kg of ket or 0.045 mg/kg midazolam over 40 min^++^	Neuroimaging: Baseline Clinical assessment: Baseline 24 h postinfusion	MADRS	Positive association between ΔMADRS and baseline left hippocampal volume
Abdallah *et al*., 2017 (94); NCT00768430					
*N*: 16 Unipolar TRD***	NO: medication-free ≥ 1 wk prior to treatment	Single IV infusion of 0.5 mg/kg of ket over 40 min^++^	Neuroimaging and clinical assessment: Baseline 24 h postinfusion	MADRS; Rs defined as posttreatment MADRS < 10	(−) Left nucleus accumbens volume following treatment in Rs only (+) Left hippocampal volume in Rs (−) Bilateral nucleus accumbens volumes after treatment in participants with ↑ left hippocampal volume, no change in participants with ↓ total or left hippocampal volume
Niciu *et al*., 2017 (98)^b^; NCT00088699					
*N*: 55 Unipolar TRD*	NO: psychotropic medication– or ECT-free ≥ 2 wk prior to infusion	Single IV infusion of 0.5 mg/kg ket over 40 min^+^	Neuroimaging and clinical assessment: Baseline 230 min, 24 h, and 1 wk postinfusion	MADRS	No significant associations between baseline hippocampal, thalamic, or amygdalar volumes and antidepressant response
Siegel *et al*., 2021 (30); NCT01179009					
*n*: 23 Unipolar TRD**, 27 HC	YES: SSRI and SNRI allowed if constant for ≥ 6 wk prior to infusion	Continuous 96-h IV infusion of ket started at 0.15 mg/kg/h at 10 AM on day 1 and titrated to tolerance twice daily to target rate of 0.6 mg/kg/h^+^	Neuroimaging: Baseline 2 wk postinfusion Clinical assessment: 2, 4, 6, and 8 wk	MADRS	Smaller pretreatment right hippocampal volume associated with better MADRS score

↑ indicates higher, ↓ indicates lower, (+) indicates increase, (−) indicates decrease, and Δ indicates change.

CA, cornu ammonis; CC, corpus callosum; CHB, Charité University Hospital Berlin; FA, fractional anisotropy; GC-ML-DG, granule cell–molecular layer–dentate gyrus; GMV, gray matter volume; IFG, inferior frontal gyrus; ILF, inferior longitudinal fasciculus; MD, mean diffusivity; PR, partial responder; PTSD, posttraumatic stress disorder; RD, radial diffusivity; SLF, superior longitudinal fasciculus; sMRI, structural magnetic resonance imaging; SNRI, serotonin and norepinephrine reuptake inhibitor; SSRI, selective serotonin reuptake inhibitor; TBM, tensor-based morphometry; UF, uncinate fasciculus; UZH, University Hospital Zurich; VBM, voxel-based morphometry; see Table 1 for other abbreviations.

a Two participant sites including CHB and UZH. CHB used a Siemens Tim Trio scanner, UZH used a Philips Achieva TX scanner.

b To pool clinical assessments from the 2 sites, HDRS scores were converted to MADRS scores and calculated as a percentage of change from baseline to follow-up.

* ≥1 failed treatment.

** ≥2 failed treatment.

*** ≥3 failed treatment.

§ HC received ket.

+ Open label.

++ Randomized placebo.

**Table 6.** Diffusion-Weighted MRI Studies of Ketamine Treatment in Depressed Populations

Sample	Other Medication	Ket Treatment	Data Collection	Depression Assessment	Summary of Findings
Sydnor *et al*., 2020 (105); NCT02544607					
*N*: 13 Unipolar TRD*	YES: stable antidepressants and psychotherapy for ≥ 28 days prior, ket naive	Single 0.5 mg/kg IV infusion over 40 min^+^	Neuroimaging: Baseline 4 h postinfusion Clinical assessment: 24 h postinfusion	HDRS	↑ Preinfusion FA in left CB–hippocampal portion and left SLF associated with improvements in HDRS (+) FA in bilateral ILF, left SLF, and right UF between pre- and postinfusion ↓ FA in CC–forceps minor and bilateral UF negatively correlated with improvement in HDRS
Vasavada *et al*., 2016 (104); NCT02165449^a^					
*n*: 10 Unipolar MDD, 15 HC	YES: 9 participants receiving concurrent antidepressant therapy	Single 0.5 mg/kg IV infusion over 40 min^+^	Neuroimaging: Baseline Clinical assessment: Baseline 24 h postinfusion	MADRS; NRs defined as ΔMADRS < 50%	R FA > NR FA in cingulum and forceps minor (−) in RD in forceps Only NRs showed significantly ↓ FA and ↓ MD in forceps minor and ↑ RD in cingulum compared with HC
Nugent *et al*., 2019 (106)^b^; NCT00088699					
*n*: 30 Unipolar TRD*, 26 HC^§^	NO: psychotropic medication–free for ≥ 2 wk	Single 0.5 mg/kg IV infusion or saline placebo^+++^	Neuroimaging: Baseline Follow-up Clinical assessment: Baseline 40, 80, 120, and 230 min postinfusion 1, 2, 3, 7, 10, and 11 days postinfusion	MADRS	(−) FA in tracts connecting right amygdala and sgACC associated with better clinical response (−) FC between left amygdala and sgACC associated with better response to ket

↑ indicates higher, ↓ indicates lower, (+) indicates increase, and (−) indicates decrease.

CB, cingulum bundle; MRI, magnetic resonance imaging; see Tables 1 and 5 for other abbreviations.

a Different cohort from the other NCT02165449 studies.

b Ket treatment results presented in the Supplement.

* ≥1 failed treatment.

§ HC received ket.

+ Open label.

+++ Randomized crossover.

**Table 7.** Magnetic Resonance Spectroscopy Studies of Ketamine Treatment in Depressed Populations

Sample	Other Medication	Ket Treatment	Data Collection	Depression Assessment	Summary of Findings
Valentine *et al*., 2011 (109)					
*N*: 10 Unipolar MDD	NO: psychotropic medication–free ≥ 2 wk prior to study	Single IV infusion of saline over 40 min followed by single IV infusion 0.5 mg/kg ket over 40 min 1 wk later^+++^	Neuroimaging: Baseline 3 h after infusion start 2 days postinfusion Clinical assessment: Baseline 60 min, 180 min, 24 h, 48 h, 72 h, 5 days, and 7 days postinfusion	HDRS-25, BDI, HARS, BPRS	No change in AANt content following ket No correlation with clinical response No cortical AANt
Milak *et al*., 2016 (110)					
*N*: 11 Unipolar MDD	NO: psychotropic medication–free ≥ 2 wk prior to study	Single intravenous infusion 0.5 mg/kg over 40 min^+^	Neuroimaging: Baseline structural MRI and ^1^H MRS 4 ^1^H MRS during infusion 1 ^1^H MRS immediately postinfusion Clinical assessment: Baseline 230 min and 24 h postinfusion	HDRS-24	Higher glutamate and GABA compared with baseline but not correlated with clinical response Norket levels at 90 min postinfusion correlated with improvement in depressive symptoms
Milak *et al*., 2020 (111)					
*N*: 38 Unipolar MDD	NO: no psychotropic medication or medication likely to interact with GABA or glutamate ≥ 2 wk, no neuroleptics ≥ 2 months, no fluoxetine ≥ 6 wk prior to study	Single intravenous infusion of 0.1, 0.2, 0.3, 0.4, or 0.5 mg/kg ket over 40 min^+^	Neuroimaging: Baseline structural MRI and ^1^H MRS 4 ^1^H MRS during infusion 1 ^1^H MRS immediately postinfusion Clinical assessment: Baseline 24 h postinfusion	HDRS-22	Glutamate correlated with clinical improvement when ket blood level not included in model GABA not correlated with clinical improvement
Evans *et al*., 2018 (112); NCT00088699					
*n*: 20 Unipolar TRD*, 17 HC^§^	NO: medication-free ≥ 2 wk prior to study	Single IV infusion 0.5 mg/kg ket or saline placebo with alternative treatment 2 wk later^+++^	Neuroimaging: Baseline 24 h postinfusion Clinical assessment: Baseline Same day as infusion	HDRS, SHAPS	Nonsignificant increase in glutamate and tNAA post ket compared with baseline and placebo

AANt, amino acid neurotransmitter; BPRS, Brief Psychiatric Rating Scale; GABA, gamma-aminobutyric acid; tNAA, *N*-acetyl aspartate summed with *N*-acetylaspartylglutamate; see Table 1 for other abbreviations.

* ≥1 failed treatment.

§ HC received ket.

+ Open label.

+++ Randomized crossover.

**Table 8.** Limitations in the Field

Limitation	Notes
Small Sample Sizes	The majority of current studies consist of small sample sizes, with the largest study in this review including 61 participants with MDD. Testing the generalizability of these findings in larger samples is crucial to improving our understanding of ketamine’s therapeutic mechanisms.
Lack of Consensus Regarding TRD Definition	There is a lack of consensus regarding the number of failed treatments that define TRD; therefore, the severity of treatment resistance within the population of each study is variable.
Heterogeneous Medication Inclusion/Exclusion Criteria	There were frequently differences in criteria for permitted concurrent antidepressant and antipsychotic medication, with some studies requiring participants to be medication-free, while others only required stable medications. It is possible that interactions between ketamine and concurrent antidepressant medication may determine treatment response (117) and should be investigated further.
Differences in Ketamine Treatment	Although the majority of the studies generally administered ketamine intravenously at 0.5 mg/kg over 40 min, there were varying treatment doses and administrative methods, and studies investigating serial ketamine treatment varied greatly in the number of treatments administered, which likely influenced treatment response.
Observations Made During Differing Windows Post Ketamine	There was significant variability regarding when neuroimaging and clinical assessments were collected following ketamine treatment, many of which may have missed the window of ketamine’s therapeutic effects.
Inconsistent Network Nomenclature	The still-maturing field of network neuroscience lacks consistent nomenclature across studies and is further complicated by fact that particular brain regions are also shared across specific networks (118,119).

This table outlines several limitations in the field to consider, particularly when trying to summarize findings and for designing future studies of ketamine antidepressant treatment.
