# Supplementary material for: Neuroimaging-Derived Biomarkers of the Antidepressant Effects of Ketamine
Source: Biol Psychiatry Cogn Neurosci Neuroimaging. Author manuscript; Available in PMC 2024 Oct 16. (PMC11483103; doi:10.1016/j.bpsc.2022.11.005)
Supplement: Supplemental Material [file NIHMS2021986-supplement-Supplemental_Material.pdf]

SUPPLEMENTARY INFORMATION

Neuroimaging-Derived Biomarkers of the Antidepressant Effects of Ketamine

Zavaliangos-Petropulu *et al.*

Supplemental Material

Methods:

Original research articles included in this review were those published in English and that used neuroimaging methods to investigate the neuroplasticity effects following ketamine treatment in human adult populations with unipolar or bipolar depression. We selectively included the following imaging approaches: resting-state or task functional MRI (fMRI), positron emission tomography (PET), structural MRI (sMRI), diffusion-weighted imaging (DWI), magnetic resonance spectroscopy (MRS), and arterial spin labelling (ASL) perfusion MRI. A literature search of the PubMed and Google Scholar databases was done using key words listed in main manuscript **Table 1.** This search revealed 51 articles published between 2011-2022.

Different imaging methods may provide unique, though complementary information concerning the brain-circuit level mechanisms associated with ketamine's therapeutic effects, and most studies have not combined data from different imaging modalities. We thus discuss findings according to the neuroimaging modality employed. We reference findings in the respective section for those analyses including measures from more than one neuroimaging approach. The majority of studies reviewed assessed imaging biomarkers a single 0.5 mg/kg dose of intravenous (IV) ketamine, though a few studies included more than one treatment or administered ketamine using different dose as noted. Changes in structure, function or connectivity were assessed at different intervals following ketamine. Many studies conducted follow-up scans 24 hours post infusion (ketamine half-life is ~2.5 hours in adults(1)) with single or repeated doses and with some including longer follow-ups. Studies focused on investigating acute response or brain imaging (<24 hours) to ketamine were not included. Response to ketamine is usually characterized as a >50% reduction in symptoms, while remission is characterized as a near normal cut-off from mood ratings such as the Hamilton Depression Rating scale (HDRS)(2) or the Montgomery-Asberg Depression Rating Scale (MADRS)(3).

Only significant findings regarding ketamine treatment in MDD patients are reported. Findings that are described as trending significance (p-value>0.05) or that do not pass multiple comparisons correction performed by the original authors are not reported.

Supplemental Table 1. Regions with changes in resting state functional connectivity described in main manuscript Figure 2 including region, direction of change, any associations with clinical assessment, and corresponding study.

ROI1	ROI2	Type	Associations with Clinical Assessment	Study
amygdala	SN	decreasing	NA	Vasavada et al. 2021
sgACC	DMN	decreasing	NA	Siegel et al. 2021
dlPFC	OBFC	decreasing	NA	Chen et al. 2019
DMN	DMN	decreasing	NA	Siegel et al. 2021
occipital	DMN	decreasing	NA	Sahib et al. 2020
cerebellum	SN	decreasing	NA	Sahib et al. 2020
cerebellum	striatum	decreasing	remitters	Sahib et al. 2020
amygdala	sgACC	decreasing	depressive symptoms	Nakamura et al. 2021
dACC	dACC	decreasing	suicidality	Chen et al. 2019
limbic	limbic	decreasing	NA	Siegel et al. 2021
limbic	CON	increasing	NA	Siegel et al. 2021
amygdala	CEN	increasing	NA	Vasavada et al. 2021
dACC	temporal	increasing	NA	Chen et al. 2019
sgACC	insula	increasing	NA	Siegel et al. 2021
DMN	insula	increasing	NA	Evans et al. 2018
DMN	Middle frontal	increasing	NA	Evans et al. 2018
DMN	postcentral	increasing	NA	Evans et al. 2018
DMN	occipital	increasing	NA	Evans et al. 2018
SMN	SMN	increasing	NA	Sahib et al. 2020
habenula	dlPFC	increasing	depressive symptoms	Rivas-Grajales et al. 2021
habenula	occipital	increasing	depressive symptoms	Rivas-Grajales et al. 2021
habenula	temporal	increasing	depressive symptoms	Rivas-Grajales et al. 2021
habenula	occipital	increasing	depressive symptoms	Rivas-Grajales et al. 2021
sgACC	SMA	increasing	depressive symptoms	Gärtner et al. 2019
sgACC	dlPFC	increasing	depressive symptoms	Gärtner et al. 2019
sgACC	aPFC	increasing	depressive symptoms	Gärtner et al. 2019
sgACC	OBFC	increasing	depressive symptoms	Gärtner et al. 2019
caudate	pgACC	increasing	anhedonia	Mkrtchian et al. 2023
dlPFC	parietal	increasing	suicidality	Chen et al. 2019
striatum	dlPFC	increasing	NA	Mkrtchian et al. 2021
caudate	vlPFC	increasing	anhedonia	Mkrtchian et al. 2022
putamen	OBFC	increasing	NA	Mkrtchian et al. 2024
sgACC	dACC	increasing	NA	Siegel et al. 2021

Supplemental Table 2. Regions with changes in BOLD-derived activity following a specific task described in main manuscript Figure 3 including paradigm, region, direction, specified task, and corresponding study.

Paradigm	Region	Direction	Task	Study
Response Inhibition	Inferior frontal cortex	-	response inhibition	Sahib et al. 2020
Response Inhibition	Dorsolateral PFC	-	response inhibition	Sahib et al. 2020
Response Inhibition	cerebellum	-	response inhibition	Sahib et al. 2020
Response Inhibition	left visual cortex	-	response inhibition	Sahib et al. 2020
Response Inhibition	superior parietal region	-	response inhibition	Sahib et al. 2020
Response Inhibition	precentral gyrus	-	response inhibition	Sahib et al. 2020
Response Inhibition	cerebellum and CEN	-	response inhibition	Loureiro et al. 2021
Incentive Processing	sgACC	-	positive and negative feedback	Morris et al. 2020
Reward Behavior	insula	+	anticipatory	Sterpenich et al. 2019
Reward Behavior	Inferior frontal cortex	+	anticipatory	Sterpenich et al. 2019
Reward Behavior	Ventral striatum	+	anticipatory	Sterpenich et al. 2019
Emotional Judgement Task	Amygdala	-	positively valanced pictures	Sterpenich et al. 2019
Emotional Judgement Task	Insula	-	positively valanced pictures	Sterpenich et al. 2019
Emotional Judgement Task	substantia nigra/ventral tegmental area	+	negative pictures	Sterpenich et al. 2019
Emotional Processing Task	medial prefrontal cortex	+	angry trials	Reed et al. 2018
Emotional Processing Task	anterior cingulate	+	angry trials	Reed et al. 2018
Emotional Processing Task	medial prefrontal cortex	-	happy trials	Reed et al. 2018
Emotional Processing Task	anterior cingulate	-	happy trials	Reed et al. 2018
Emotional Processing Task	parahippocampal gyrus	-	angry trials	Reed et al. 2018
Emotional Processing Task	amygdala	-	angry trials	Reed et al. 2018
Emotional Processing Task	amygdala	-	positive and negative faces	Loureiro et al. 2021
Emotional Processing Task	cingulate gyrus	-	angry trials	Reed et al. 2018
Emotional Processing Task	precuneus	-	angry trials	Reed et al. 2018
Emotional Processing Task	middle frontal gyrus	-	angry trials	Reed et al. 2018
Emotional Processing Task	cingulate gyrus	+	happy trials	Reed et al. 2018
Emotional Processing Task	precuneus	+	happy trials	Reed et al. 2018
Emotional Processing Task	middle medial frontal gyrus	+	happy trials	Reed et al. 2018
Emotional Perception Faces	caudate	+	happy > neutral	Murrough et al. 2015

Supplemental Table 3. Resting state fMRI study demographics

Study	Demographics	Age (years)	Sex	Illness Details
Abdallah et al. 2017	N[A]: 22 Unipolar TRD***, 47 HC (29 HC from Cohort A, 18 HC from Cohort B)	• Cohort A TRD: 44 ± 2.3<br>• Cohort A HC: 44 ± 1.8<br>• Cohort B HC: 28 ± 0.9	• Cohort A TRD: 10F/12M<br>• Cohort A HC: 13F/16M<br>• Cohort B HC: 18M	Average Age of Depression Onset: 23.4 ± 2.2 years
Abdallah et al., 2017	N: 18 Unipolar TRD**, 25HC	• TRD: 43 ± 2.2<br>• HC: 39 ± 3.1	• TRD: 8F/10M<br>• HC: 12F/13M	Average Lifetime Illness: 13.0 ± 3.4 years
Abdallah et al., 2018	N: 58 Unipolar MDD<br><br>n=19 given saline placebo<br>n=21 given ketamine<br>n=20 given lanicemine	• Saline: 25.7<br>• Ketamine: 27.1<br>• Lanicemine: 26.7	• Saline: 10F/8M<br>• Ketamine: 12F/7M<br>• Lanicemine: 12F/7M	-
Evans et al., 2018	N: 33 Unipolar TRD*, 25 HC	• TRD: 36 ± 10<br>• HC: 33 ± 10	• TRD: 20F/13M<br>• HC: 15F/10M	-
Chen et al., 2019	N: 48 Unipolar TRD**<br><br>n=16 given 0.5mg/kg<br>n=16 given 0.2mg/kg<br>n=16 given placebo	• 0.5 mg/kg: 43.3 ± 11.9<br>• 0.2mg/kg: 44.4 ± 10.8<br>• Placebo: 49.9 ± 8.1	• 0.5mg/kg: 11F/5M<br>• 0.2mg/kg: 11F/5M<br>• Placebo: 13F/3M	-
Gärtner et al., 2019	N[B]: 24 Unipolar TRD**	44.4 ± 11.8	14F/10M	-
Chen et al., 2020	N: 48 Unipolar TRD**, 40 HC<br><br>Rs=18<br>NRs=30	• Rs: 43.0 ± 12.3<br>• NRs: 48.0 ± 10.7<br>• HC: 42.4 ± 7.4	• Rs: 14F/4M<br>• NRs: 21F/9M<br>• HC: 22F/26M	Average Lifetime Illness:<br>• Rs: 10.7 ± 8.5 years<br>• NRs: 11.1 ± 8.4 years
Kraus et al., 2020	N: 28 Unipolar TRD*, 22 HC	• MDD: 33.9 ± 10.6<br>• HC: 36 ± 9.7	-	Average Lifetime Illness: 19.7 ± 12.1 years
Sahib et al., 2020	N: 61 Unipolar TRD**, 40 HC	• TRD: 38.96 ± 10.7<br>• HC: 32.87 ± 12.7	• TRD: 30F/31M<br>• HC: 24F/16M	Average Lifetime Illness: 22.81 ± 12.42 years
Zhuo et al., 2020	N: 38 Bipolar TRD**	43.1 ± 5.3	16F/22M	Average Lifetime Illness: 100.5 ± 23.2 months
Mkrtchian et al., 2020	N: 33 Unipolar TRD*, 25 HC	• TRD: 36 ± 9.54<br>• HC: 34 ± 10.97	• TRD: 18F/15M<br>• HC: 14F/11M	-
Nakamura et al., 2021	N: 14 unipolar TRD***, 1 bipolar TRD*	45.9 ± 12.5	9F/6M	Average Lifetime Illness: 1.3 ± 2.1 years
Siegel et al., 2021	N: 23 Unipolar TRD**, 27 HC	40.0 ± 14.05	10F/13M	-
Rivas-Grajales et al., 2021	N: 35 Unipolar TRD**	42.2 ± 13.9	16F/19M	-
Vasavada et al., 2021	N: 44 Unipolar TRD**, 50 HC	• MDD: 38.2 ± 10.9<br>• HC: 32.3 ± 11.9	• MDD: 18F/26M<br>• HC: 27/23	Average Lifetime Illness: 20.2 ± 12.1 years
Zhang et al. 2020	N: 35 Unipolar TRD	33.9±8.22	22F/6M	Average Lifetime Illness: 21.50±3.25 months

Supplemental Table 4. Task-based fMRI study demographics

Study	Demographics	Age (years)	Sex	Illness Details
Sahib et al., 2020	N: 47 Unipolar TRD**, 32 HC	• TRD: 38.6 ± 10.6<br>• HC: 34.8 ± 13.5	• MDD: 19F/28M<br>• HC: 18F/14M	Average Lifetime Illness: 21.7 ± 12.3 years
Loureiro et al., 2021	[C]N: 46 Unipolar TRD** (T1 N=42, T2 N=39, T3 N=40), 32 HC	• T1: 39.24 ± 10.96<br>• T2: 39.23 ± 10.41<br>• T3: 40.3 ± 11.16<br>• HC: 35.16 ±13.54	• T1: 26F/16M<br>• T2: 23F/16M<br>• T3: 22F/18M<br>• HC: 9F/23M	Average Lifetime Illness (years):<br>• T1: 22.35 ± 13.32<br>• T2: 20.60 ± 12.21<br>• T3: 22.09 ± 13.24
Murrough et al., 2015	N: 18 Unipolar TRD**, 20 HC	• TRD: 28.1 ± 13.8<br>• HC: 35.0 ± 8.9	• TRD: 8F/10M<br>• HC: 9F/11M	Average Lifetime Illness: 24.2 ± 15.7 years
Sterpenich et al., 2019	N: 10 Unipolar TRD**	median(range): 51(38-58)	6F/4M	Average Lifetime Illness median(range): 21(4-29) years
Morris et al., 2020	N:<br>• Study 1: 28 Unipolar MDD, 20 HC<br>• Study 2: 16 Unipolar TRD**	• Study 1:<br>-MDD: 36.5 ± 11<br>-HC: 37.8 ± 9.4<br>• Study 2: 44.7 ± 11.4	• Study 1:<br>-MDD: 14F/14M<br>-HC: 7F/13M<br>• Study 2: 9F/7M	-
Reed et al., 2018	N: 33 Unipolar TRD*, 26 HC	• TRD: 36.1 ± 9.7<br>• HC: 33.9 ± 10.4	• MDD: 21F/12M<br>• HC: 15F/10M	-
Reed et al., 2019	N: 33 Unipolar TRD*, 24 HC	• MDD: 35.9 ± 9.8<br>• HC: 34.4 ± 10.7	• MDD: 20F/13M<br>• HC: 15F/9M	-
Loureiro et al., 2020	N: 27 Unipolar TRD**, 31 HC	• TRD: 37.3 ± 10.8<br>• HC: 34.5 ± 13.5	• TRD: 11F/16M<br>• HC: 19F/13M	Average Lifetime Illness: 19.31 ± 12.73 years
Downey et al 2016	N: 56 unipolar MDD<br>Ketamine: N=21<br>Lanicemine: N=20<br>Placebo: N=19	• Ketamine=27.<br>• Lanicemine=26.7<br>• Placebo=25.7	• Ketamine=13F/8M<br>• Lanicemine=12F/8M<br>• Placebo=11F/8M	-
McMillan et al. 2020	N: 26 unipolar MDD	30.2 ± 8.2	13F/13M	<5 years N=6<br>5-10 years N=7 >10 years N=11
Stippl et al. 2021	N: 16 Unipolar MDD	44.19 ± 14.92	10F/6M	-

Supplemental Table 5. PET study demographics

Study	Demographics	Age (years)	Sex	Illness Details
Estrelis et al., 2018	N: 14 Unipolar MDD, 13 HC	• MDD: 35.6 ± 13.6<br>• HC: 33.1 ± 13.1	• MDD: 8F/6M<br>• HC: 9F/4M	Age of Onset: 25.1 ± 12.0 years
Tiger et al., 2020	N: 30 Unipolar TRD* (10 placebo, 10 ketamine)	• Placebo: 37.1<br>• Ketamine: 39.2	• Placebo: 6F/4M<br>• Ketamine: 8F/12M	-
Lally et al., 2014	N: 36 Bipolar (I or II) TRD*	46.69 ± 11.09	21F/15M	Age of Onset: 17.89 ± 7.39 years
Lally et al., 2015	N: 20 TRD**	48.29 ± 12.84	-	Age on Onset: 19.92 ± 11.84 years
Chen et al., 2018	N: 24 Unipolar TRD***<br><br>0.5mg/kg: n=8<br>0.2mg/kg: n=8<br>placebo=8	• 0.5mg/kg: 51.13 ± 13.59<br>• 0.2mg/kg: 49.75 ± 11.08<br>• Placebo: 46.25 ± 8.14	• 0.5mg/kg: 8F/0M<br>• 0.2mg/kg: 5F/3M<br>• Placebo: 5F/3M	-
Ortiz et al. 2015	N: 29 Bipolar TRD*	46.9 ± 10	-	-

Supplemental Table 6. ASL study demographics

Study	Demographics	Age (years)	Sex	Illness Details
Sahib et al., 2020	N: 22 Unipolar TRD*, 18 HC	TRD: 35.27 ± 9.95<br>HC: 36.11 ± 14.5	TRD: 6F/16M<br>HC: 10F/8M	Average Lifetime Illness: 18.91 ± 11.91 years
Gärtner et al., 2022	N: 21 Unipolar MDD	43.6 ± 12.17	12F/8M	-
Gonzalez et al., 2020	N: 11 Unipolar TRD**	47.7 ± 11.9	3F/8M	-

Supplemental Table 7. sMRI study demographics

Study	Demographics	Age (years)	Sex	Illness Details
Gallay et al., 2021	N: 30 MDD with chronic suicidality	45.7 ± 14.2	16F/14M	-
Herrera-Melendez et al., 2021	N: 33 Unipolar TRD** (23 CHB, 10 UZH)	• CHB:49.13 ± 12.24<br>• UZH: 42.1 ± 12.99	• CHB: 12F/11M<br>• UZH: 6F/4M	-
Dai et al., 2020	N: 21 Unipolar MDD (10 with comorbid PTSD), 29 HC	• MDD: 35.8 ± 2.7<br>• HC: 32.9 ± 2.3	• MDD: 12F/9M<br>• HC: 15F/14M	-
Zhou et al., 2020	N: 44 Unipolar TRD**	35.2 ± 12.2	28F/16M	Average Lifetime Illness: 76.6 ± 77.3 months
Zhou et al., 2020	N: 44 Unipolar TRD**, 45 HC	• MDD: 35.2 ± 12.2<br>• HC: 33.0±11.2	• MDD: 28F/16M<br>• 24F/21M	Average Lifetime Illness: 76.6 ± 77.3 months
Abdallah et al., 2015	N: 13 Unipolar TRD***	46.6 ± 2.6	6F/8M	Average Lifetime Illness: 18.9 ± 2.5 years
Abdallah et al., 2017	N: 16 Unipolar TRD***	45.9 ± 2.7	7F/9M	-
Niciu et al., 2017	N: 55 Unipolar TRD*	41.5 ± 12.9	28F/27M	Average Lifetime Illness: 23.5 ± 12.4 years
Siegel et al., 2021	N: 23 Unipolar TRD**, 27HC	40.0 ± 14.05	10F/13M	-

Supplemental Table 8. dMRI study demographics

Study	Demographics	Age (years)	Sex	Illness Details
Sydnor et al., 2020	N: 13 Unipolar TRD*	42.0 ± 13.9	8F/5M	Average Length of Current Depressive Episode: 108.8 ± 125.1 months
Vasavada et al., 2016	N: 10 Unipolar MDD (6 Rs, 4 NRs), 15 HC	• NRs: 50.7 ± 12.4<br>• Rs: 45.7 ± 12.2<br>• HC: 45.7 ± 11.0	• NR: 4M<br>• R: 2F/4M<br>• HC: 5F/10M	Average Length of Current Depressive Episode:<br>• NRs: 222 ± 242.4 months<br>• Rs: 117.6 ± 135.6 months
Nugent et al., 2019	N: 30 Unipolar TRD*, 26 HC	• MDD: 35 ± 9.4<br>• HD: 34 ± 10.3	• MDD: 16F/14M<br>• HC: 16F/10M	Average Lifetime Illness: 19 ±9 .7 years

Supplemental Table 9. MRS study demographics

Study	Demographics	Age (years)	Sex	Illness Details
Valentine et al., 2011	N: 10 Unipolar MDD	41.7 ± 12	6F/4M	Average Lifetime Illness: 21.2 ± 17.4 years
Milak et al. 2016	N: 11 Unipolar MDD	38.8 ± 12.8	8F/3M	Average Lifetime Illness: 16.6 ± 9.4 years
Milak et al. 2020	N: 38 Unipolar MDD	38.6±11.2	23F/15M	
Evans et al., 2018	N: 20 Unipolar TRD*, 17 HC	TRD: 36.2 ± 2.5<br>HC: 34.7 ± 2.9	TRD: 12F/8M<br>HC: 12 F/5M	Average Lifetime Illness: 21 ± 2.7 years

Supplemental Table 10. Resting State fMRI Neuroimaging Parameters

Study	Scanner Manufacturer	Scanner Strength	Voxel Size	Seed Based or Data Driven	Atlas Used
Abdallah et al. 2017	Philips	3T	3.4 x 3.4 x 4 mm	GBC	Desikan-Killiany
Abdallah et al., 2017	Philips	3T	2.2 x 2.2 x 3.25 mm	GBC<br>Additional Seeds:<br>subgenual anterior cingulate cortex, dorsolateral PFC, posterior cingulate	Desikan-Killiany
Abdallah et al., 2018	Philips	3T	3 × 3 × 2.5 mm	GBC	-
Evans et al., 2018	General Electric	3T	3.75 x 3.75 x 3.5 mm	Seed Based<br>Seeds:<br>posterior cingulate cortex, insula, anterior cingulate cortex	-
Chen et al., 2019	General Electric	3T	3.5 × 3.5 × 3.5 mm	Seed Based<br>Seeds:<br>dorsal anterior cingulate cortex, dorsolateral PFC, medial PFC	-
Gärtner et al., 2019	Siemens<br>Philips	3T	Siemens:<br>3 x 3 x 3 mm<br><br>Philips:<br>2.75 x 2.75 x 4 mm	Seed Based:<br>Seeds:<br>subgenual anterior cingulate cortex, dorsolateral PFC, posterior cingulate cortex, hippocampus	Harvard-Oxford
Chen et al., 2020	General Electric	3T	3.5 x 3.5 x 3.5 mm	Seed Based<br>Seeds:<br>striatum	Oxford-GSK-Imanova
Kraus et al., 2020	General Electric	3T	3.75 x 3.75 x 3.5 mm	GBC<br>Additional Seeds:<br>Prefrontal cortex	-
Sahib et al., 2020	Siemens	3T	2 x 2 x 2 mm	Data Driven	-
Zhuo et al., 2020	General Electric	3T	3.4 x 3.4 x 4 mm	gFCD	-
Mkrtchian et al., 2020	General Electric	3T	3.75 x 3.75 x 3.5	Seed Based<br>Seeds:<br>ventral striatum, dorsal caudate, dorsal caudal putamen, ventral rostral putamen	-
Nakamura et al., 2021	General Electric	3T	1.875 x 1.875 x 4 mm	Seed Based<br>Seeds:<br>Ventral precuneus, amygdala	Harvard-Oxford
Siegel et al., 2021	Siemens	3T	4 x 4 x 4 mm	Seed Based<br>Seeds:<br>Amygdala,<br>anterior hippocampus,<br>posterior hippocampus,<br>nucleus accumbens	Gordon-Lauman parcellation
Rivas-Grajales et al., 2021	Siemens	3T	2.18 x 2.18 x 2.18 mm	Seed Based<br>Seeds:<br>Habenula	MNI
Vasavada et al., 2021	Siemens	3T	2 x 2 x 2 mm	Seed Based<br>Seeds:<br>Amygdala, hippocampus	Harvard-Oxford

Supplemental Table 11. Task-based fMRI neuroimaging parameters

Study	Scanner Manufacturer	Scanner Strength	Voxel Size (fMRI)	Analysis	Atlas Used
Sahib et al., 2020	Siemens Prisma	3T	2 x 2 x 2 mm	Voxel-based and ROI	Desikan-Killiany
Loureiro et al., 2021	Siemens Prisma	3T	2 x 2 x 2 mm	PPI (cerebellum and cortex)	LSN atlas
Murrough et al., 2015	Philips Achieva X-Series	3T	2.2 x 2.2 x 2.2 mm	Seed (caudate)	MNI
Sterpenich et al., 2019	Siemens Trio	3T	3.2 x 3.2 x 3.2 mm	ROI (amygdala, insula, anterior cingulate cortex, orbitofrontal cortex, ventral striatum)	MNI
Morris et al., 2020	Siemens MAGNETOM Skyra	3T	2.1 x 2.1 x 2.1 mm	Seed (sgACC)	Broadmann
Reed et al., 2018	General Electric Signa HDx	3T	3.75 x 3.75 x 3.5 mm	Voxel-based	-
Reed et al., 2019	General Electric Signa HDx	3T	3.75 x 3.75 x 3.5 mm	Voxel-based	-
Loureiro et al., 2020	Siemens Prisma	3T	2 x 2 x 2 mm	Voxel-based	-
Downey et al. 2016	Philips Achieva & Siemens TimTrio	3T	3 x 3 x 3 mm	Voxel-based	-
McMillan et al. 2020	Siemens Skyra	3T	3 x 3 x 3 mm	Voxel-based and ROI (sgACC)	MNI
Stippl et al. 2021	Philips Achieva, Siemens Trio	3T	Philips Achieva: 2.75 x 2.75.4 mm<br><br>Siemens Trio: 3 x 3 x 3 mm	ROI (dorsolateral prefrontal cortex, posterior cingulate cortex, dorsomedial prefrontal cortex, amygdala, insula, pregenual anterior cingulate cortex)	Neurosynth

Supplemental Table 12. PET neuroimaging parameters

Study	Scanner Manufacturer	Scanner Strength	Voxel Size (T1)	Analysis	Atlas Used
Estrelis et al. 2018	GE Signa Advantage	3T	1.02 x 1.02 x 1.00 mm	ROI (34 regions)	described in Ogden et al. 2007(4)
Tiger et al. 2020	GE MR750	3T	-	ROI (hippocampus, ventral striatum, ACC, dorsal brainstem)	Desikan-Killiany, MNI
Lally et al. 2014	GE Signa	3T	0.9 x 0.9 x 1.2 mm	ROI (ventral striatum and orbitofrontal cortex)	MNI
Lally et al. 2015	GE Signa	3T	0.9 x 0.9 x 1.2 mm	ROI (ventral striatum and orbitofrontal cortex) and Whole Brain	MNI
Chen et al. 2018	GE Discovery 750	3T	1 x 1 x 1 mm	ROI (dACC) and Whole Brain	-
Ortiz et al. 2015	GE Signa	3T	0.9 x 0.9 x 1.2 mm	ROI (amygdala, hippocampus)	MNI

Supplemental Table 13. ASL neuroimaging parameters

Study	Scanner Manufacturer	Scanner Strength	Voxel Size (ASL)	Seed Based or Data Driven	Atlas Used
Sahib et al. 2020	Siemens Prisma	3T	2.5 x 2.5 x 2.5 mm	ROI and Whole Brain	Desikan-Killiany
Gärtner et al. 2021	Siemens and Philips	-	Siemens:<br>3 x 3 x 5 mm<br><br>Philips:<br>3 x 3 x 6 mm	Whole Brain	-
Gonzalez et al. 2020	Siemens Allegra	3T	4 x 4 x 7.5 mm	Whole Brain	-

Supplemental Table 14. sMRI neuroimaging parameters

Study	Scanner Manufacturer	Scanner Strength	Voxel Size (T1)	Analysis	Atlas
Herrera-Melendez et al., 2021	Siemens Tim Trio<br><br>Philips Achieva TX	3T	1 × 1 × 1 mm	VBM	Hammers brain atlas
Gallay et al., 2021	Siemens Skyra	3T	-	VBM	-
Dai et al., 2020	Siemens Tim Trio scanner later upgraded to a Siemens Prisma	3T	1 × 1 × 1 mm	TBM	automated anatomical labeling (AAL3) atlas
Zhou et al., 2020	Philips Achieva X-series	3T	1 × 1 × 1 mm	ROI	Desikan-Killiany
Zhou et al., 2020	Philips Achieva X-series	3T	1 × 1 × 1 mm	ROI	Freesurfer hippocampal subfield atlas
Abdallah et al., 2015	Siemens Trio	3T	1 × 1 × 1 mm	ROI	Desikan-Killiany
Abdallah et al., 2017	GE EXCITE	3T	0.9 x 0.9x 1.5mm	ROI	Desikan-Killiany
Niciu et al., 2017	GE scanners	3T	1 × 1 × 1 mm	ROI	Desikan-Killiany
Siegel et al., 2021	Siemens Trio TIM scanner	3T	4 x x 4 mm	ROI	Desikan-Killiany

Supplemental Table 15. dMRI neuroimaging parameters

Study	Scanner Manufacturer	Scanner Strength	Voxel Size (DWI)	Directions	Analysis	Atlas
Sydnor et al. 2020	Siemens MAGNETOM PRISMAFit	3T	2x2x2mm	64 b=1000<br>10 b=0	TBSS	IIT WM Atlas
Vasavada et al. 2016	Siemens Allegra	3T	2.5x2.5x2.5	55 b=1000<br>10 b=0	TBSS	JHU WM Atlas
Nugent et al. 2019	GE	3T	-	6 b=0<br>12 b=300<br>42 b=1100	Along tract statistics	Desikan-Killiany

Supplemental Table 16. MRS neuroimaging parameters

Study	Scanner Manufacturer	Scanner Strength	Voxel Size (MRS)	Region	Spectra
Valentine et al. 2011	Bruker	4T	-	Occipital cortex	GABA, Glx, NAA, NAAG, creatine, phosphocreatine, myoinositol, choline, phosphorylcholine, glycerophosphorylcholine, schylloinositol
Milak et al. 2016	GE Electric Signa EXCITE	3T	1 x 1 x 1 mm	Medial prefrontal cortex	GABA, Glx
Milak et al. 2020	GE Electric Signa EXCITE	3T	1 x 1 x 1 mm	Ventral medial prefrontal cortex	GABA, Glx
Evans et al. 2018	Siemens Magnetom	7T	1 x 1 x 1 mm	pgACC	glutamate, glutamine, glutathione, GABA, NAA, NAAG, choline, and creatine

Supplemental References

1. Autry AE, Adachi M, Nosyreva E, Na ES, Los MF, Cheng P-F, *et al.* (2011): NMDA receptor blockade at rest triggers rapid behavioural antidepressant responses. *Nature* 475: 91–95.
2. Hamilton M (1960): A rating scale for depression. *J Neurol Neurosurg Psychiatry* 23: 56–62.
3. Montgomery SA, Asberg M (1979): A new depression scale designed to be sensitive to change. *Br J Psychiatry* 134: 382–389.
4. Ogden RT, Ojha A, Erlandsson K, Oquendo MA, Mann JJ, Parsey RV (2007): In vivo quantification of serotonin transporters using [(11)C]DASB and positron emission tomography in humans: modeling considerations. *J Cereb Blood Flow Metab* 27: 205–217.
